# Analysis of cancer-associated fibroblasts in cervical cancer by single-cell RNA sequencing

**DOI:** 10.18632/aging.205353

**Published:** 2023-12-28

**Authors:** Shuang Wen, Xuefeng Lv, Pengxiang Li, Jinpeng Li, Dongchun Qin

**Affiliations:** 1 Reproductive Center, The First Affiliated Hospital of Zhengzhou University, Zhengzhou, Henan, China; 2 Department of Laboratory Medicine, The Third Affiliated Hospital of Zhengzhou University, Zhengzhou, Henan, China; 3 Henan Provincial Chest Hospital, Zhengzhou, Henan, China; 4 Department of Laboratory Medicine, The First Affiliated Hospital of Zhengzhou University, Zhengzhou, Henan, China

**Keywords:** cervical cancer, single-cell RNA sequencing, tumor microenvironment, cancer-associated fibroblasts, tumor heterogeneity

## Abstract

Objective: Since scRNA-seq is an effective tool to study tumor heterogeneity, this paper intends to reveal the differences of cervical cancer in patients at the individual cell level by scRNA-seq, and focus on the biological functions of cancer-associated fibroblasts (CAFs) in cervical cancer, facilitating the provision of a new interpretation of the heterogeneity of the microenvironment of cervical cancer, and an in-depth exploration of the pathogenesis of cervical cancer as well as pursuit of effective means of treatment intake.

Methods: 3 cervical cancer specimens were collected by clinical surgery for single-cell RNA sequencing. Cell suspensions of fresh cervical cancer tissues were prepared, and cDNA libraries were created and sequenced on the machine. Furthermore, the sequencing data were analyzed using bioinformatics, including descending clustering of cells, identification of cell populations, mimetic time series analysis, inferCNV, cell communication analysis, and identification of transcription factors.

Results: A total of 9 cell types were identified, encompassing T cells, epithelial cells, smooth muscle cells, CAFs, endothelial cells, macrophages, B cells, lymphocytes, and plasma cells. CAFs were further divided into three cell subtypes, named type1 cells, type2 cells, and type3 cells. With key transcription factors for the three cells, TCF21, ZC3H11A, and MYEF2 obtained, this research revealed the communication relationship between CAFs and several other cells, and found an important role of CAFs in the MK signaling pathway.

Conclusions: scRNA-seq technology contributed to exploring the tumor heterogeneity of cervical cancer more deeply, and also further gaining insight into the biological functions of CAFs in cervical cancer.

## INTRODUCTION

Cervical cancer (CC) is the most common family of tumors and one of the leading causes of cancer-related deaths in women worldwide [1]. With the popularization of HPV vaccination and early screening, the incidence of cervical cancer has declined significantly, and surgery, radiation, and chemotherapy have been commonly used for patients with cervical cancer [2]. However, the three-year and five-year survival rates for patients with advanced and metastatic cervical cancer remain suboptimal [1, 3]. It is generally believed that CC tumors exhibit a high degree of heterogeneity in the intratumor and microenvironment, causing cancer progression in the course of disease development, thus leading to treatment failure [4].

One of the important sources of tumor heterogeneity is molecular variation within tumor subclones or even between individual cells. Cancer is a highly heterogeneous disease with phenotypic diversity, often influenced by abnormalities at epigenetic, transcriptional, and protein levels that interact in the tumor microenvironment [4, 5]. Single-cell RNA sequencing allows for the precise and rapid determination of gene expression patterns in thousands of individual cells [6]. Analysis of individual cells provides more meaningful insights into cellular behavior than analysis of aggregates, helping to identify potential heterogeneity among cell populations and providing new perspectives on tumor heterogeneity.

There are few reports on single-cell sequencing studies of cervical cancer. Despite the increasing adoption of single-cell sequencing technology, its application in oncology is still limited to a few types. This has led to the fact that the intratumoral transcriptional heterogeneity of cervical cancer, common cancer in women, is largely unknown. In this study, the scRNA-seq analysis of cervical cancer was performed by collecting cervical cancer tissues to reveal the characteristics of different cell populations in cervical cancer, providing new insights into the heterogeneity of cervical cancer at the single-cell level. Cancer-associated fibroblasts (CAFs) play an important role in tumors, and can suppress the function of immune cells by secreting various cytokines or metabolites, thus promoting tumor development, invasion, and metastasis [7]. As a result, the present study focused on the biological functions of CAFs in the development of cervical cancer.

## MATERIALS AND METHODS

### Selection of research subjects

This research selected surgically resected tumor tissues from three patients with cervical cancer hospitalized at the First Affiliated Hospital of Zhengzhou University for single-cell sequencing. This study was approved by the Ethics Committee of the First Affiliated Hospital of Zhengzhou University (ethics number: 2018-KY-28), and informed consent was obtained from patients. Besides, single-cell sequencing data were attained from GSE168652 in the Gene Expression Omnibus (GEO) database for one case of cervical cancer paracancer tissue (GSM5155197) [8].

### Preparation of fresh single-cell suspensions

After surgery, the excised tissues were placed in lyophilized tubes containing tissue protection solution pre-cooled at 4° C and washed with DPBS pre-cooled at 4° C and repeated 2-3 times to remove the residual tissue protection solution. Then, with a small amount of digestion solution added, the tissue was cut into small pieces and placed in centrifuge tubes (50 ml) containing 5 mL of digestion solution and digested by shaking in a constant temperature water bath at 37° C. After digestion, with the digest passed through a pre-wetted 40 μm cell sieve, the filtrate was collected into a new 50 mL centrifuge tube. 2 mL of pre-cooled erythrocyte lysate at 4° C was added to the cell sediment, gently blown and mixed, and incubated at room temperature for 3 min. Further, 10 μL of the blown cell suspension was taken and gently mixed with 10 μL of 0.4% Typan blue. Finally, 10μL of the obtained mixture was used to be quickly added to the Countess® II Cell Counting plate.

### Library construction and sequencing

The prepared cell suspensions, together with 10×Barcode gel beads and oil, were added to Chromium Chip B microplates, and the beads and cells with Cell Barcode were encapsulated in droplets by microfluidic techniques. These cell-encapsulated droplets were collected and the cells in the droplets were lysed to form GelBeads-in-emulsion (GEM) through a microfluidic “double-cross” crossover system. GEM was then reversely transcribed using a PCR instrument, and RNA in the cells was reversely transcribed into the first strand of cDNA with the barcode Unique Molecular Identifier (UMI) information using the reverse transcription primers on the gel beads. The first strand of cDNA was further purified utilizing magnetic beads for RT-PCR amplification (12 cycles of amplification). After cDNA amplification was completed, it was fragmented using enzymes and the optimal fragment was screened with magnetic beads to construct a cDNA library by sequencing primers by PCR. The labeled cDNA library mix was sequenced on the Illumina NovaSeq platform. The sequencing was performed by the end-pairing procedure for double-end sequencing, and Illumina provided software to manipulate the sequencing process and to analyze the real-time data generated by sequencing.

### Quality control of sequencing data

Sample quality control was conducted using Cell Ranger, the official software of 10x genomics, which integrates STAR software internally [9]. The reads were first compared to the reference genome to obtain quality control results such as high-quality cell count, gene count, and genomic matching rate in the raw data. Subsequently, the quality of each sample was assessed. The Seurat (version 4.3.0) [10] software package was adopted to further QC the experimental data based on the initial QC of Cell Ranger to exclude data that were multicellular, bicellular, or unbound on cells. Cells with a total gene count of less than 7000 and more than 200 and a mitochondrial UMI percentage of less than 10% were retained as high-quality cells. Double cells were removed for downstream analysis with the aid of the DoubletFinder (version 2.0.3) [11] software. Eventually, three cases of cervical cancer sequencing data and one case of single-cell sequencing data from paracancerous tissue were merged, with Harmony (version 0.1.1) [12] employed to remove batch effects during the merging process.

### Data downscaling and clustering

The first 50 principal components were selected as statistically significant components for subsequent analysis, and the dimensionality reduction results were visualized in two dimensions using t-distributed random neighborhood embedding (t-SNE) plots [13, 14]. The research utilized the single-cell sequencing cell type annotation software Single R [15] which annotates cell types by calculating the correlation between the single-cell reference expression profile dataset and the cell expression profile to be identified and annotates the cell to be identified as a cell type with the highest correlation with the reference dataset. Further, the cell types were artificially annotated based on Marker genes, in combination with results from published literature (Supplementary File 1).

### Detection of copy number variation in tumor cells

InferCNV [16] can be used to identify large-scale chromosomal copy number variations (CNV) in tumor single-cell RNA-Seq data, including amplifications and deletions of whole or large segments of chromosomes. This approach determines the intensity of gene expression by comparing the gene expression of each tumor cell with that of a reference cell (normal cells). Therefore, this study adopted the InferCNV (version 1.10.1) software package to detect CNVs in epithelial cells, identifying real tumor cells. The control group was 1 cluster of epithelial cells derived from normal tissue.

### Cellchat analysis of intercellular communication

CellChat refers to an open-source R package (https://github.com/sqjin/CellChat), which can be applied for the analysis and visualization of scRNA-seq data intercellular communication (version1.6.1) [17]. CellChat is capable of characterizing different intercellular communication by a variety of learning methods including pattern recognition methods [18] and can combine the interaction between gene expression and signaling ligands and receptors to construct a probabilistic model of cell-to-cell communication. Such an analytical model can accurately elaborate on specific signals played by each cell population. Consequently, CellChat was used in this paper to analyze the communication between the nine cell types that were identified.

### Proposed time series analysis of CAFs subtypes

The clustering of CAFs was downscaled and time series analysis of their subpopulations was proposed to visualize cell movement trajectories and map kinetic expression heat maps.

### Transcription factor analysis of CAFs subtypes

PySCENIC [19] is an implementation for SCENIC [20] (Single Cell Regulatory Network Inference and Clustering) on python, which can infer transcription factors, gene regulatory networks, and cell types from single-cell RNA-seq data. Compared to SCENIC (which is based on the R language), pySCENIC offers a dramatic increase in computational speed. Since pySCENIC scales easily to multicore clusters when running on a computer, tens of thousands of cells can be analyzed in a relatively short period. This computing process is mainly achieved through the Dask framework of distributed computing. Hence, pySCENIC was utilized for transcription factor analysis of CAFs subsets in this study. Additionally, to distinguish cell subtypes more efficiently, the Ag20 (Average per 20 cells) [21] method was chosen for single-cell data and repeated three times to eliminate the error caused by random sampling.

### Availability of data and materials

The dataset supporting the conclusions of this article is included within the article. The relevant codes can be found in Supplementary File 2.

## RESULTS

### Patient information

Basic information collected from 3 patients with cervical cancer is as follows (Table 1).

**Table 1:** Basic patient information.

	**Patient 1**	**Patient 2**	**Patient 3**
Age	27	47	35
Gender	Female	Female	Female
Tumor Type	Cervical squamous carcinoma	Cervical squamous carcinoma	Cervical squamous carcinoma
HPV infection	16 (+)	16 (+)	16 (+)
Chemotherapy	Yes	Yes	Yes
Clinical Staging	IIa	IIa	IIb
Drug resistance	None	None	None

### Results of dimensionality reduction clustering for single-cell data

**Figure 1:**
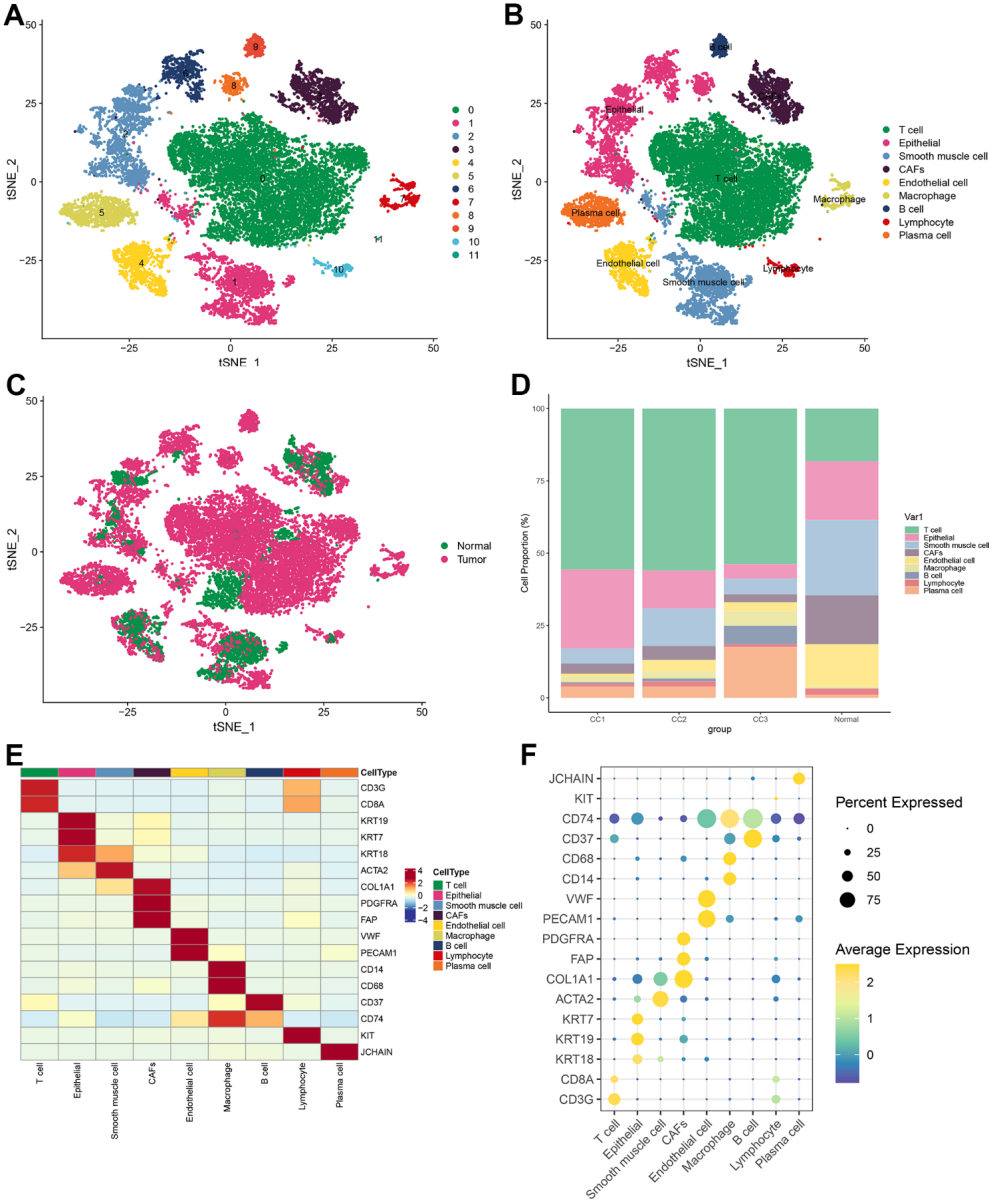
**Visualization of tSNE for cell descending clustering.** (**A**) tSNE plot of 12 cell clusters; (**B**) tSNE plot of 9 cell types; (**C**) tSNE plot of normal and tumor cells; (**D**) Proportion of each cell type in different samples; (**E**) Heat map of marker gene expression for 9 types of cells; (**F**) Bubble map of marker gene expression for 9 types of cells.

Visualization of the clustering results by t-SNE revealed that all cells from 3 tumor samples and 1 normal sample from the database were clustered into 12 cell populations (Figure 1A). To clarify the type of each cell population, the marker gene was obtained for each cell population. Each cell subpopulation based on the above-identified marker genes was then annotated using Single R. The annotation results were based on marker genes from the Cell Marker database, combined with cell marker genes from the published literature for more detailed identification of each cell subpopulation. The results demonstrated that 12 cell subpopulations were annotated into 9 cell types: T cells, epithelial cells, smooth muscle cells, CAFs, endothelial cells, macrophage cells, B cells, lymphocytes, and plasma cells (Figure 1B). In the t-SNE plots according to tissue type, it was observed that immune cells (T cells, B cells, and plasma cells) occupied a large part of the cells in the tumor tissue (Figure 1C). The proportion of different cells in each sample was statistically analyzed and plotted as a histogram. It was found that the proportion of T cells was significantly higher in tumor samples than that in normal samples; while in normal samples, smooth muscle cells and endothelial cells were more predominant (Figure 1D). Marker genes of various cells were well distinguished for cell types, and each marker gene was abundantly expressed in its corresponding cell type (Figure 1E). Specifically, CD8A was highly expressed in T cells; KRT19 was highly expressed in epithelial cells; ACTA2 was highly expressed in smooth muscle cells; PDGFRA, FAP, and COL1A1 were highly expressed in CAFs; CD74 was highly expressed in macrophage cells; CD37 was highly expressed in B cells; KIT was highly expressed in lymphocytes; JCHAIN was highly expressed in plasma cells (Figure 1F).

### GSEA pathway enrichment analysis for each cell type

**Figure 2:**
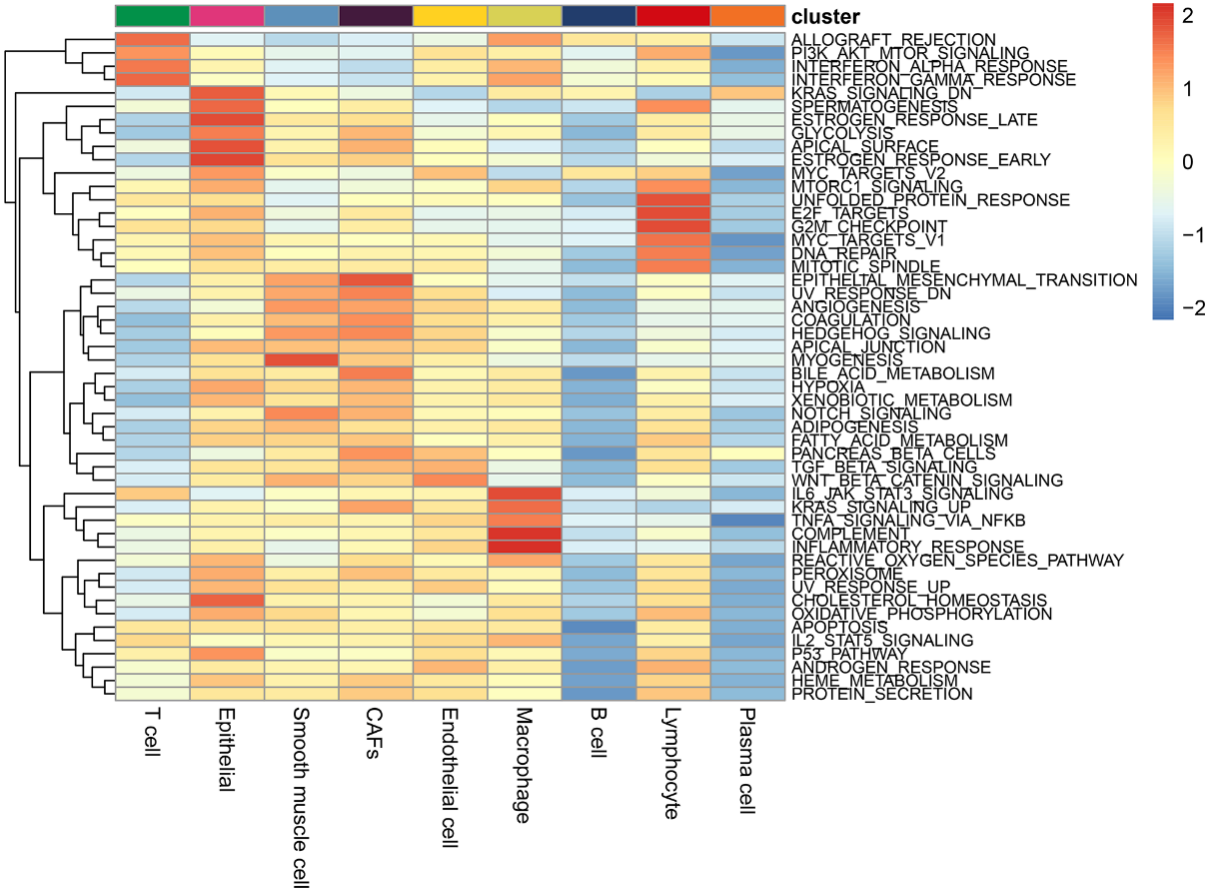
**Enrichment pathways in each cell type.** The colours represent the enrichment of the pathways, with the pathway enrichment increasing from blue to red.

ALL_OGRAFT_REJECTION, PIK_AKT_mTOR signaling pathway, α interferon response, γ interferon response were enriched in T cells; The IL6_JAK_STAT3 signaling pathway, complement, inflammatory response, and upregulation of KRAS signaling pathway, and TNFa/NF_kb signaling pathway were enriched in macrophages; The pathways of epithelial and mesenchymal transition and coagulation were significantly enriched in CAFs; Wnt/beta-catenin signaling pathway was significantly enriched in endothelial cells (Figure 2).

### CNV profile in tumor

**Figure 3:**
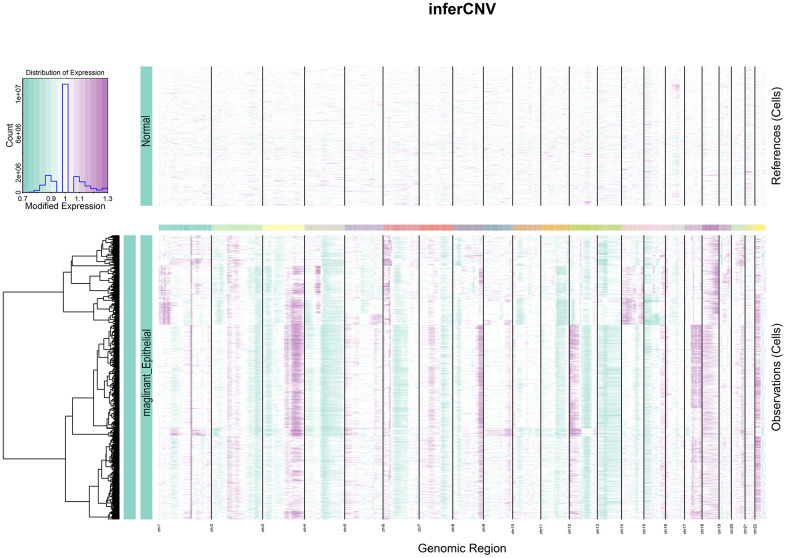
**InferCNV heat map.** The colours represent the copy number, which gradually increases from green to purple.

Heterogeneity among tumors is often caused by copy number variation [22]. Cervical squamous carcinoma, the most common type of cervical cancer, is a squamous cell carcinoma of epithelial origin. This study used the gene copy number of normal epithelial cells as control and analyzed the degree of malignancy of epithelial-derived cells by the inferCNV algorithm. Epithelial cell clusters were selected from the cell population and separated according to tumor and normal tissues. As observed from the figure, a significantly higher copy number level occurred in the tumor group than in the normal group (Figure 3).

### Intercellular communication network

**Figure 4:**
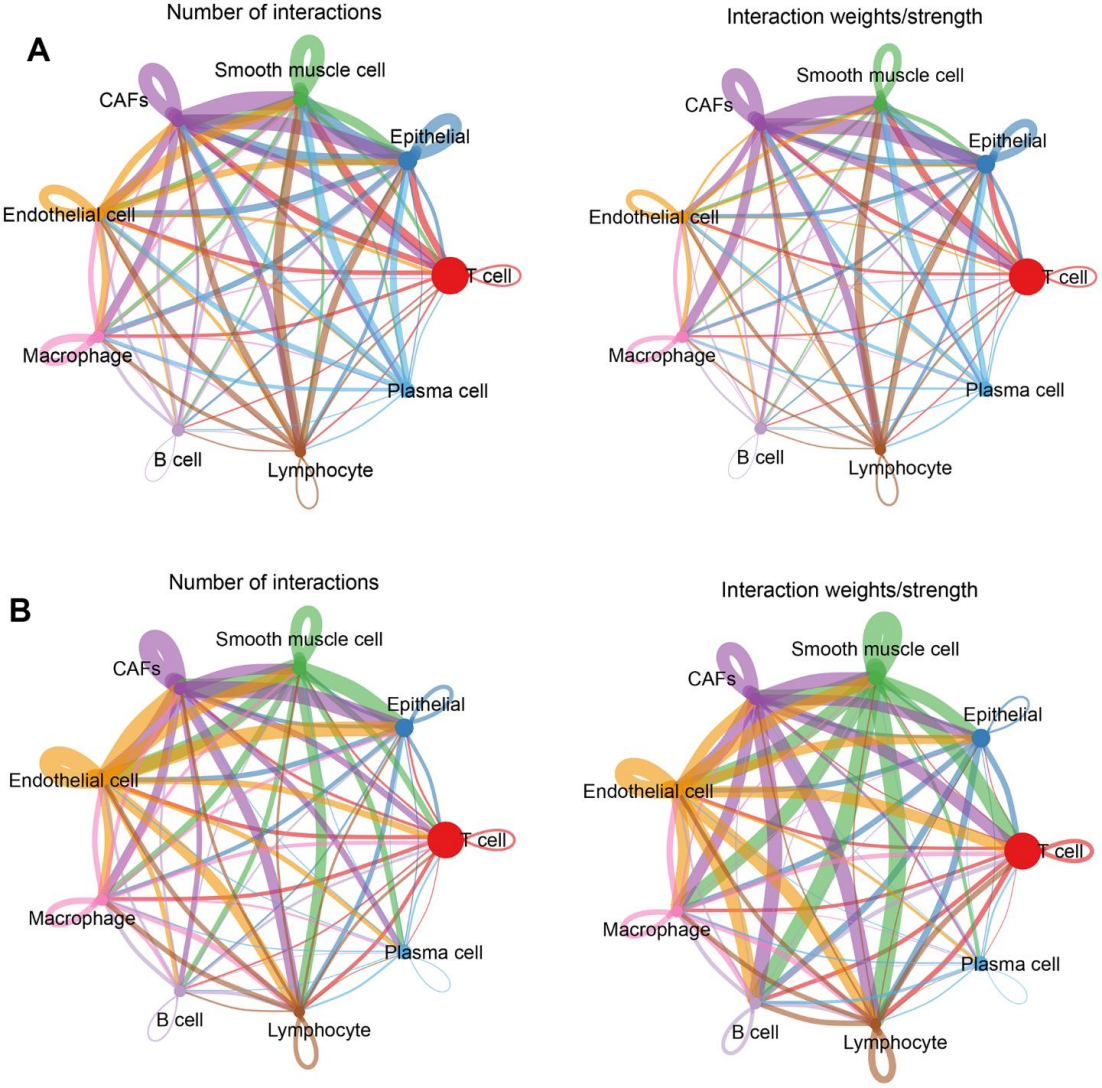
**Cellular communication networks.** (**A**) Number of interactions and intensity of action of different cell types in the normal group; (**B**) Number of interactions and intensity of action of different cell types in the tumor group. The thickness of the line refers to the number of interactions and the strength of the effects.

Intercellular receptor ligands and signaling pathways were calculated using CellChat to analyze intercellular interactions. For analysis, the cells were separated into two groups according to normal and tumor tissues and the associations between different cell types in the two subgroups were calculated separately. In normal tissues, CAFs and other cells presented more interactions and stronger effects (Figure 4A); while in tumor tissues, endothelial cells, CAFs, smooth muscle cells, and other cells had more interactions and stronger effects (Figure 4B). This means that there was more than one cell involved in the development of a tumour.

### Changes in intercellular communication in the tumor group relative to the normal group

**Figure 5:**
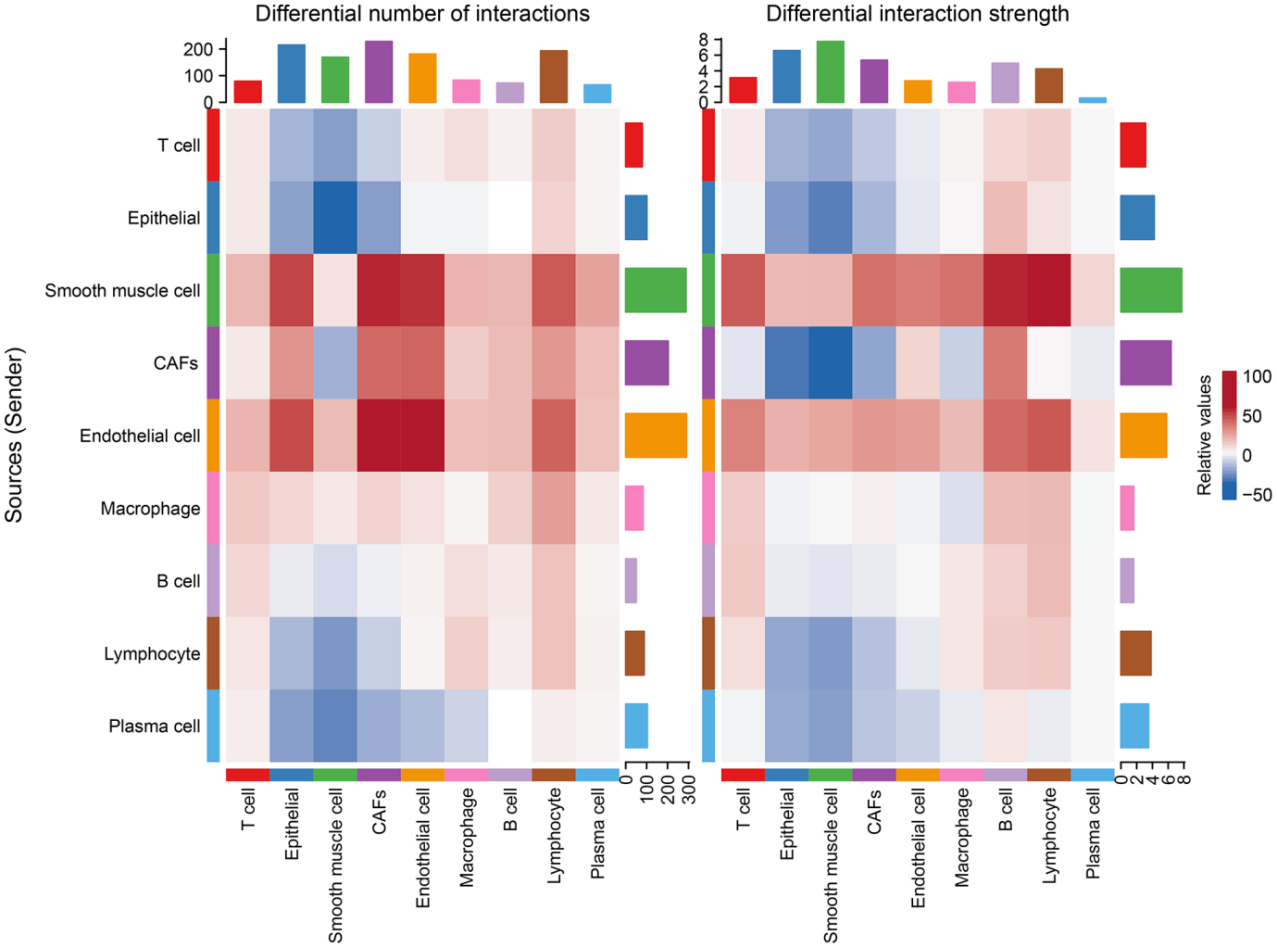
Changes in the number of interactions and intensity of the effects of different cell types in the tumor versus normal group, with blue representing weakening and red representing intensification.

After differential analysis of cellular communication between the tumor and normal groups, it was found that, relative to the normal group, endothelial cells, smooth muscle cells, CAFs, and macrophages were enhanced in the number of interactions with other cells in the tumor group, while the intensities of the effects of endothelial cells, smooth muscle cells, and other cells were improved, along with largely positive correlations (Figure 5).

### Communication between CAFs and other cells

**Figure 6:**
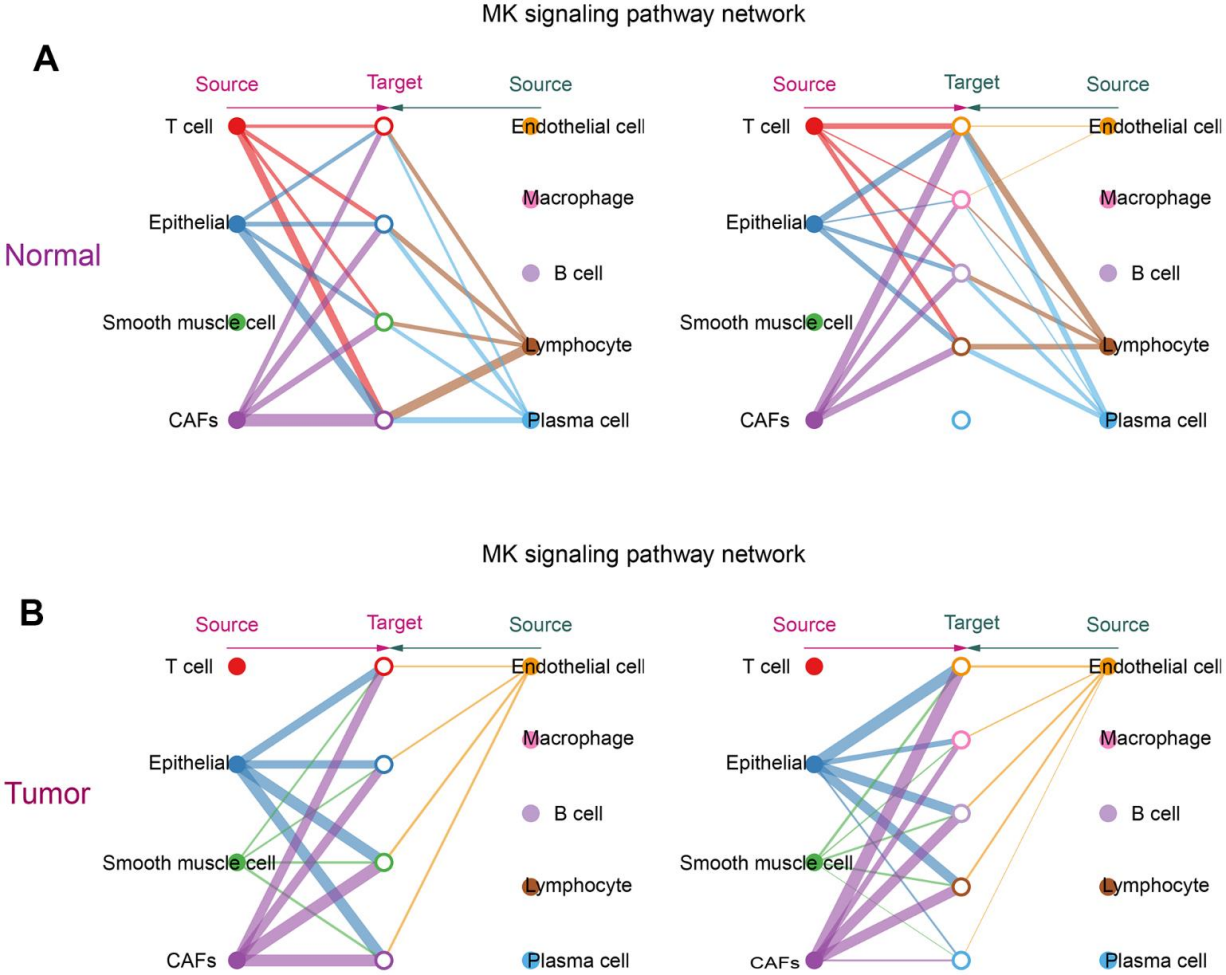
**Communication of CAFs with other cells in the MK signaling pathway.** (**A**) Hierarchical diagram of the MK signaling pathway in the communication network of the normal group of cells; (**B**) Hierarchical diagram of the MK signaling pathway in the communication network of the tumor group of cells. This diagram consists of two parts: the left and right parts highlight the autocrine and paracrine signals of CAFs and several other cells, respectively. The solid and hollow circles represent the source and target, respectively. Different colors of the circles represent different cells, and the width of the connecting lines represents the communication probability. The color of the connecting lines is consistent with the source of the signal.

From the obtained ligand-receptor pairs and signaling pathways, the MK (Midkine) pathway was chosen as the object of analysis. In both tumor and normal groups, CAFs could be the main source of MK action on several other cells in the MK pathway. This suggested that CAFs were the main mediators in the MK pathway, acting as gatekeepers for cell-cell communication. (Figure 6A, 6B).

### Role of CAFs in the Midkine signaling pathway

**Figure 7:**
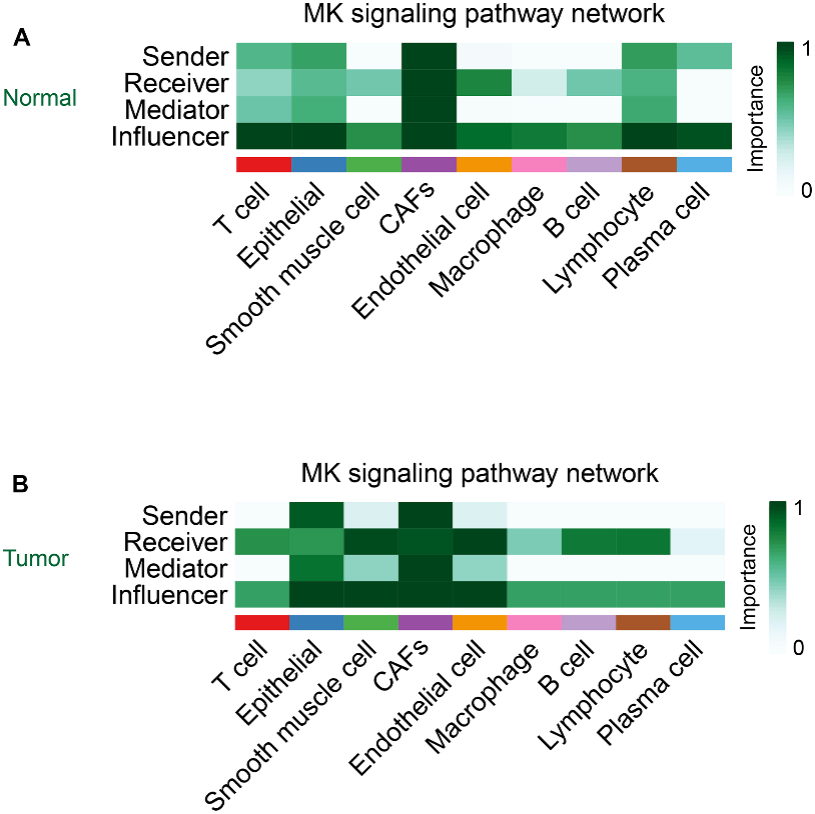
The role of CAFs in the MK signaling pathway in the normal group (**A**) and the tumor group (**B**).

Cellchat takes the centrality metric previously used in graph theory for social network analysis to identify the major sources, targets, key mediators, and key influencers in cellular communication [17]. In contrast to other cells, CAFs acted as important signal sources, targets, mediators, and influencers in the MK signaling pathway in both normal and tumor groups (Figure 7A, 7B).

### Defining new subtypes of CAFs

**Figure 8:**
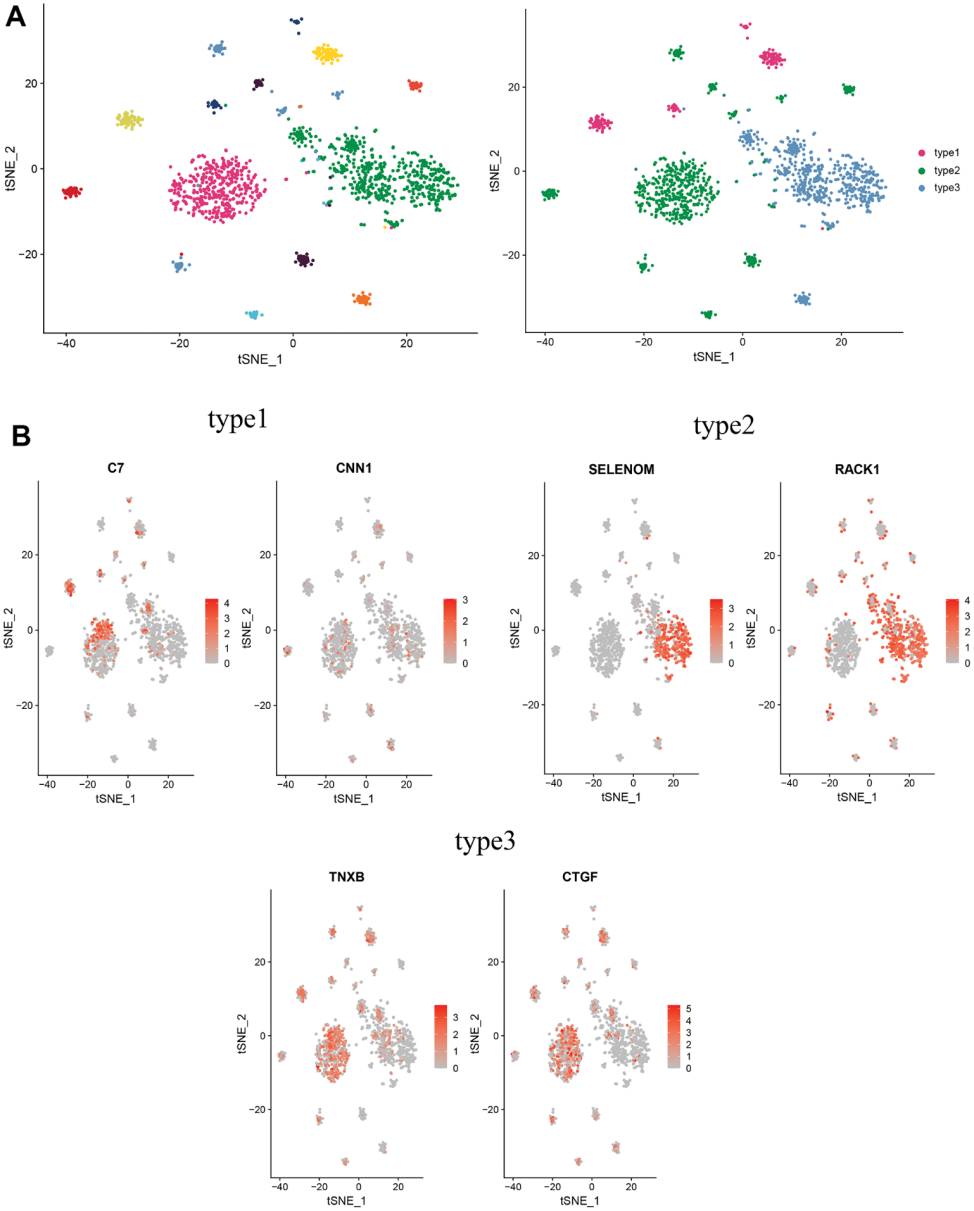
**Distinguishing new subtypes of CAFs.** (**A**) tSNE plot of CAFs with reduced dimensional clustering; (**B**) tSNE plot of maker genes for 3 cell subpopulations.

To further investigate the biological functions of CAFs in cervical cancer, CAFs were re-clustered to distinguish different subgroups. CAFs were re-clustered into 11 clusters (Figure 8A), with cells identified according to the marker gene of each cluster. Three of these clusters, 4, 5, and 6, with high expression of C7 and CNN1, were named type1 cells; two clusters, 0 and 8, with high expression of SELENOM and RACK, were named type2 cells; and several clusters, 1, 2, 3, 7, 9 and 10, with high expression of CTGF and TNXB, were named type3 cells (Figure 8B).

### Analysis of the cell developmental trajectories of each subpopulation of CAFs

**Figure 9:**
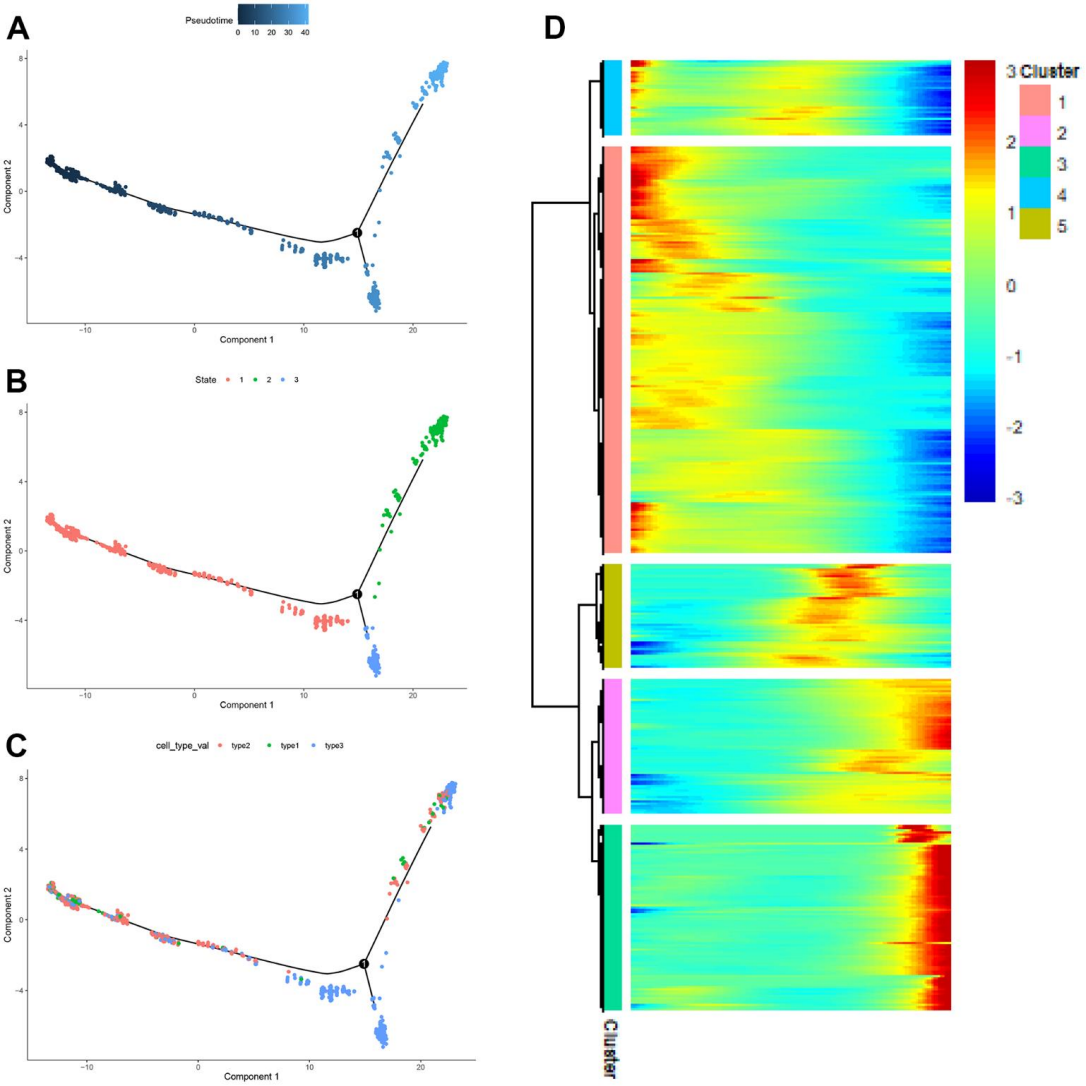
**The pseudo-time trajectory of CAFs.** (**A**–**C**) The pseudo-time trajectory of CAFs with gene expression profiles derived from Monocle 2, with each point representing a single cell; (**D**) Heat map of gene expression changes.

It could be seen from Figure 9A that the three subpopulations were divided into three developmental states. Figure 9B showed the timeline of cell differentiation, with the shades of color representing early and late developmental stages. Figure 9C introduces the developmental stages in which different subpopulations of CAFs were located. As could be seen from Figure 9C, most type2 cells were at the early developmental stage of CAFs, most type3 cells were at the middle developmental stage of CAFs, and most type1 cells were at middle and late developmental stages of CAFs. The expression heat map of genes was drawn according to the developmental trajectory, and these genes were clustered into 5 modules (Figure 9D). From the heat map, it was observed that cluster1, cluster5, and cluster3 represented the early, middle, and late stages of CAFs development, respectively.

### KEGG pathway enrichment analysis of genes in cluster 1, cluster 5, and cluster 3

**Figure 10:**
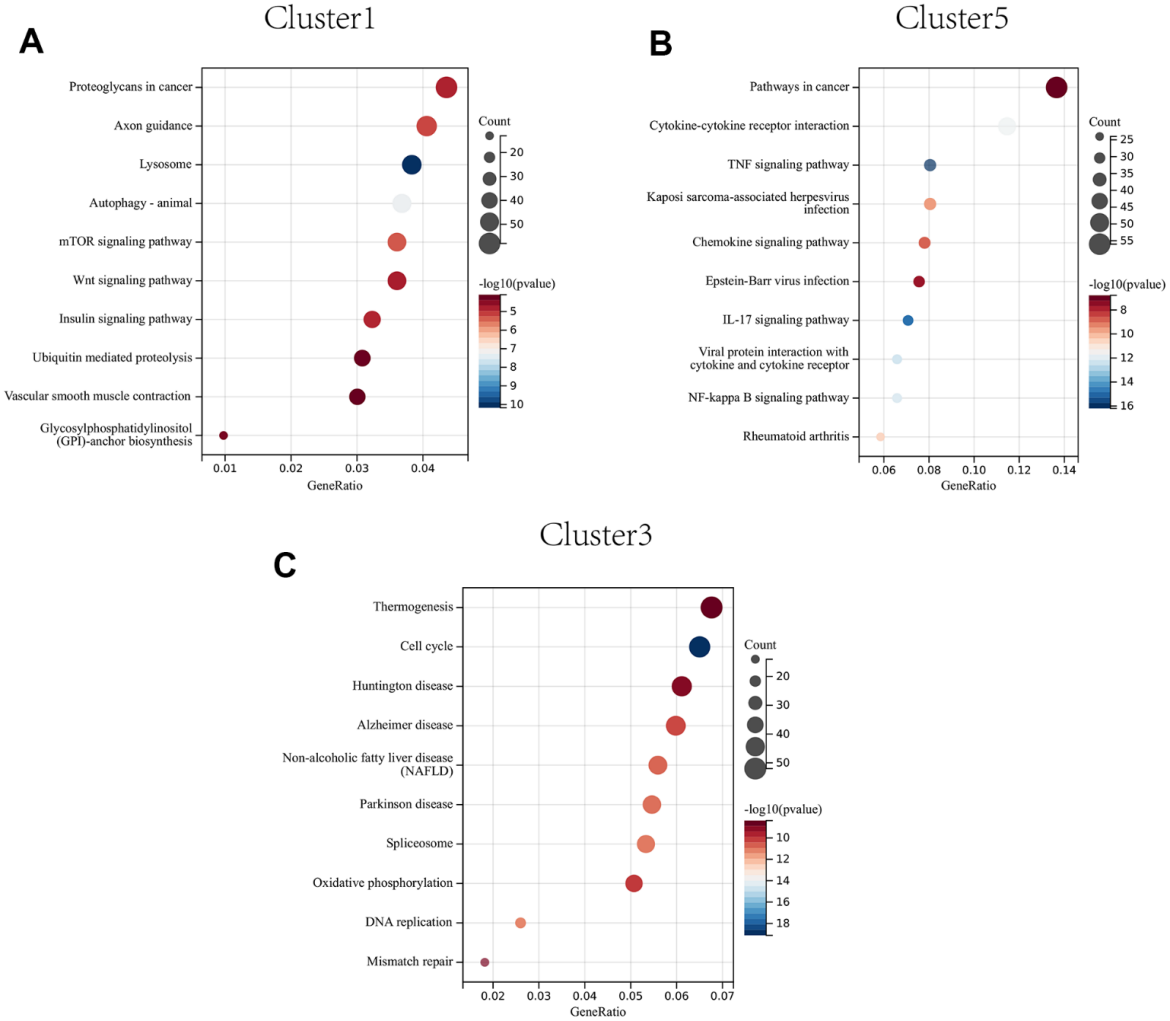
KEGG enrichment analysis of CAFs developing in early (**A**) to mid (**B**) to late (**C**) stages.

The proteoglycans in cancer, axon guidance, and lysosome were significantly enriched in CAFs at early developmental stages (cluster 1) (Figure 10A); The pathways in cancer, IL-17 signaling pathway, and cytokine-cytokine receptor interactions were significantly enriched in CAFs at mid-developmental stages (cluster 5) (Figure 10B); The cell cycle, DNA replication and mismatch repair pathways were significantly enriched in CAFs at the late developmental stage (cluster 3) (Figure 10C).

### Transcription factor prediction of CAFs subtypes

**Figure 11:**
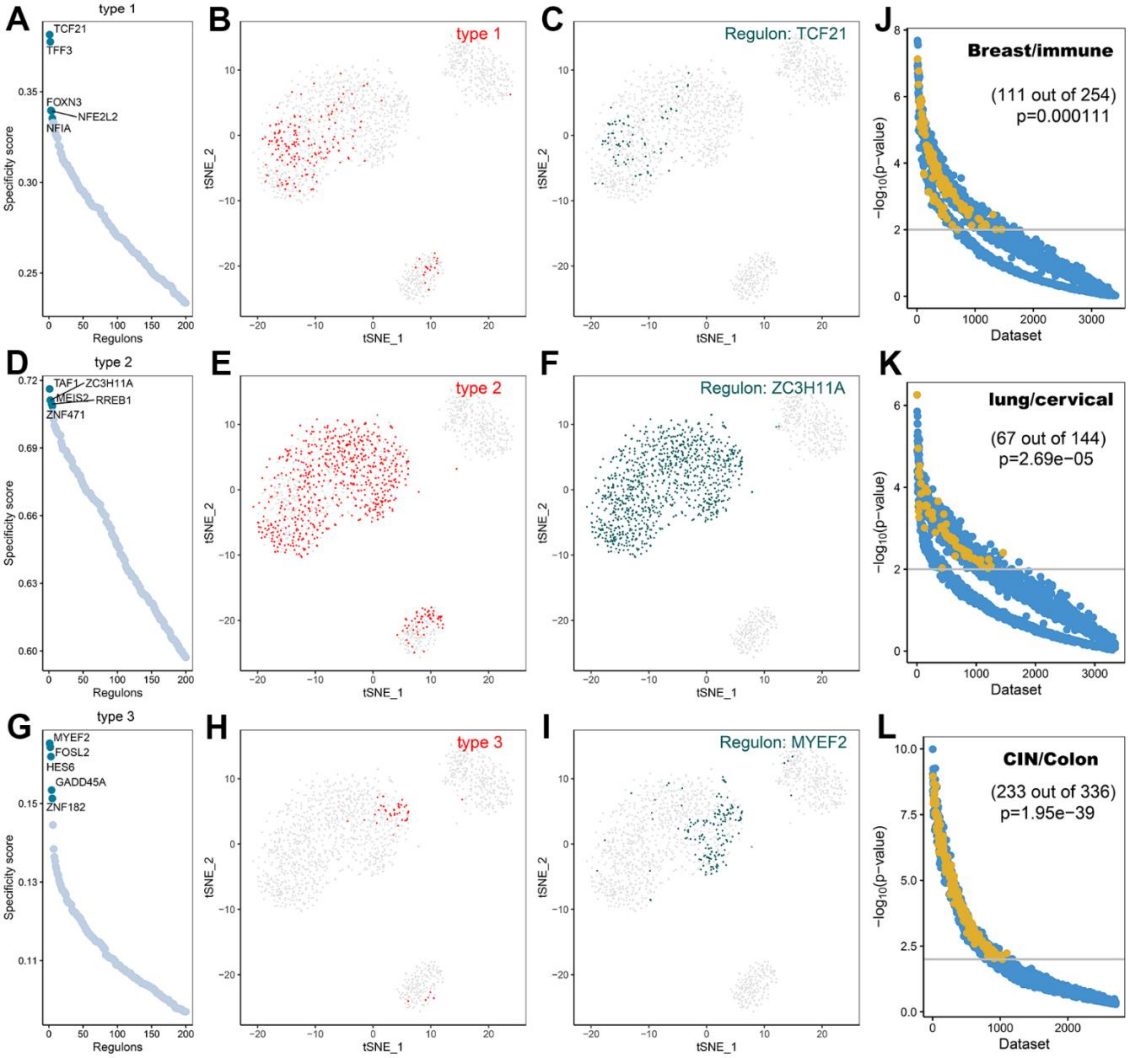
**Transcription factor prediction of CAFs subtypes.** (**A**) Ranking of transcription factors of type1 cells based on transcription factor specificity score (RSS); (**B**) Highlighting type1 in the t-SNE plot (indicated by red dots); (**C**) Binary conversion of transcription factor activity scores for transcription factors with higher RSS TCF21 (Z-Score normalization for all samples with a 2.5 dividing line to convert values to 0 and 1), highlighting in t-SNE plots (indicated by green dots); (**D**) Ranking of transcription factors of type2 cells based on transcription factor specificity score; (**E**) Highlighting type2 in the t-SNE plot (indicated by red dots); (**F**) Binary conversion of transcription factor activity scores for transcription factors with higher RSS ZC3H11A, highlighting in t-SNE plots (indicated by green dots); (**G**) Ranking of transcription factors of type3 cells based on transcription factor specificity score; (**H**) Highlighting type3 in the t-SNE plot (indicated by red dots); (**I**) Binary conversion of transcription factor activity scores for transcription factors with higher RSS MYEF2, highlighting in t-SNE plots (indicated by green dots); (**J**) Co-expression results of TCF21 and its target genes in different GEO datasets retrieved using SEEK.; (**K**) Co-expression results of ZC3H11A and its target genes in different GEO datasets retrieved using SEEK; (**L**) Co-expression results of MYEF2 and its target genes in different GEO datasets retrieved using SEEK. The x-axis represents different datasets and the y-axis represents the statistical significance of the correlation between target genes in each dataset. Functionally related and significantly correlated (p < 0.01) datasets are indicated by yellow dots.

A comprehensive network analysis of pySCENIC results was performed to systematically identify key transcription factors of the cells. The activity of each transcription factor associated with three fibroblast subtypes was evaluated, leading to the definition of a transcription factor specificity score (RSS) based on the Jensen-Shannon scatter [23]. The positively regulated transcription factors with the highest RSS values were then selected and their functional properties were further examined. Therefore, TCF21, TFF3, and FOXN3 were identified as type1 cell-specific transcription factors (Figure 11A) and their specificity was validated using tSNE plots (Figure 11B, 11C); TAF1, ZC3H11A, MEIS2, RREB1, and ZNF471 were identified as type2 cell-specific transcription factors (Figure 11D) and their specificity was validated using tSNE plots (Figure 11E, 11F); FOSL2, MYEF2, GADD45, IES6, ZNF1 were identified as type3 cell-specific transcription factors (Figure 11G), and their specificity was also validated utilizing tSNE plots (Figure 11H, 11I). Furthermore, the accuracy of these transcription factor predictions was systematically evaluated using two methods, namely SEEK [24] and CoCiter [25]. SEEK is a method based on mining public datasets and CoCiter is an approach for mining the literature. Notably, SEEK should be employed first to filter a dataset in which a transcription factor and its target gene present co-expression. Usually, the functions of these co-expressed genes tend to reflect the functions of that class of cells. Consequently, it is effective to search the headings of the dataset to obtain the functions of the cells being studied. Secondly, since descriptions related to functionally relevant genes tend to appear in abstracts in the literature, this research identified the functions of these genes and corresponding cell types in the literature abstracts by CoCiter analysis.

For type1 cells, SEEK was used to search for GEO datasets where TCF21 and its target genes were significantly co-expressed. Among the more than 3000 datasets detected, those associated with type1 cells were ranked high (Figure 11J, p<0.05). The two terms of “Breast” and “immune” from the descriptions of the datasets were extracted as a description of the function of type1 cells. For type2 cells, in the dataset of more than 3000 ZC3H11A detections showing co-expression with their target genes, most of those associated with type2 cells were ranked high (Figure 11K, p < 0.05). The two terms of “Lung” and “cervical” from the descriptions associated with the dataset were extracted. For type3 cells, SEEK was adopted to search the GEO dataset for significant co-expression of MYEF2 and its target genes. The higher-ranked ones were associated with type3 cells (Figure 11L, p < 0.05). The two terms of “CIN” and “Colon” were extracted from the descriptions of the dataset as the most frequently occurring terms for type3 cells.

### Modularization of transcription factors

**Figure 12:**
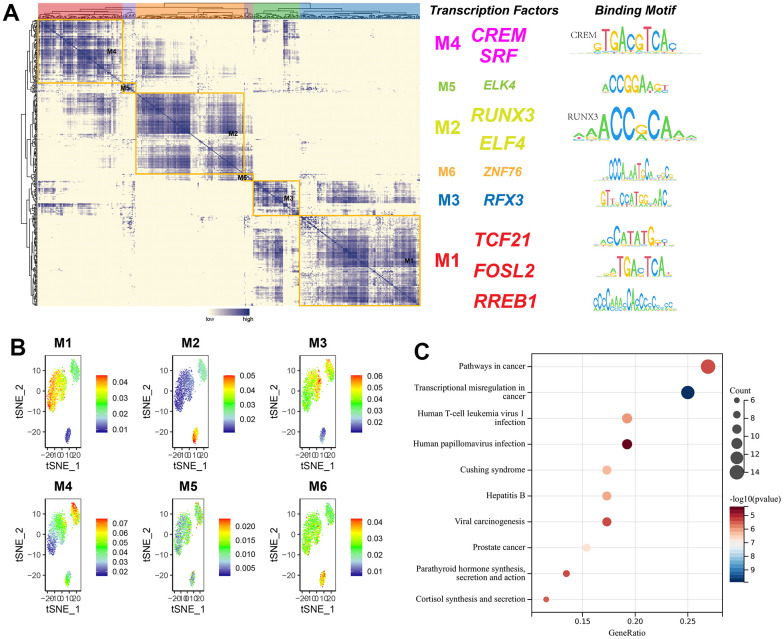
**The modularity of transcription factors and pathway enrichment analysis.** (**A**) Identification of transcription factor modules based on transcription factor ligand specificity indices, with representative transcription factors selected, and binding sequences of transcription factors plotted; (**B**) tSNE plot of the average transcription factor activity score for each module; (**C**) KEGG pathway enrichment analysis of genes in module 1.

Transcription factors usually interact with each other to regulate gene expression. To describe this mode of action more systematically, this research compared transcription factor activity scores (RAS) based on the Connection Specificity Index (CSI) [26], allowing transcription factors with similarities to cluster into one category. The results indicated that these transcription factors were mainly distinguished into six modules. The binding sequences of transcription factors were subsequently mapped by selecting representative transcription factors in each module based on their average activity scores. The representative transcription factors of M1 included TCF21, FOSL2, and RREB1, which were three subtypes of cell-specific transcription factors (Figure 12A). When the average activity fraction of each module was expressed using tSNE, it was found that each module occupied a different region, along with the presence of a complementary relationship between all highlighted regions of these modules (Figure 12B). KEGG pathway enrichment analysis of transcription factors in M1 revealed that significantly enriched pathways were connected with cancer, transcriptional misregulation in cancer, viral oncogenic effects, and human papillomavirus infection (Figure 12C).

## DISCUSSION

Cervical cancer, the fourth most common female tumor, threatens the health of women. In recent years, the heterogeneity of many tumors and their microenvironment has been well described, but the heterogeneity of cervical cancer and the reasons for its treatment failure have not been more comprehensively explained. Herein, the heterogeneity of cervical cancer was investigated with the help of scRNA-seq technology, revealing the biological roles of different cells in cervical cancer by classifying cervical cancer cells into different types, which provided good evidence for the study of cervical cancer heterogeneity. At the same time, a more in-depth study of CAFs in cervical cancer was also conducted, revealing the heterogeneity of CAFs and their biological transformation during the development of cervical cancer. Our study not only provided a more specific description of the tumor microenvironment heterogeneity in cervical cancer, but also offered new ideas for targeted therapy in cervical cancer.

A total of nine cell types in cervical cancer were identified by scRNA-seq, including T cells, epithelial cells, smooth muscle cells, CAFs, macrophages, endothelial cells, B cells, lymphocytes, and plasma cells. Subsequently, pathway enrichment analysis of the nine cell types using GSEA was performed, indicating that some characteristic pathways, such as “coagulation”, were significantly enriched in CAFs, which have been shown to be involved in the extracellular mesenchymal organization, cell migration regulation and angiogenesis [27]. CAFs were also divided into three subtypes and had three developmental states, with pathways such as “metabolic pathway” and “Wnt signaling pathway” being significantly enriched in CAFs at early developmental stages and Wnt/beta-catenin signaling pathway being significantly enriched in CAFs at early developmental stages. The Wnt/beta-catenin signaling pathway regulates various important embryonic and somatic processes, such as cell differentiation, organ development, and the formation of homeostasis within tissues [28]. Considering that CAFs are mainly transformed by fibroblasts under the stimulation of growth factors and that normal fibroblasts can also acquire the phenotype of CAFs by communicating with cancer cells, early CAFs exhibit similar functions as normal fibroblasts; The “TNF signaling pathway” and “IL-17 signaling pathway” are the most important pathways, which are significantly enriched in CAFs in middle developmental stages (cluster 2), and TNF (tumor necrosis factor) serves as the role of immunomodulator [29]. The IL-17 signaling pathway is associated with autoimmune diseases and cancer progression [30]. This suggests that CAFs become involved in immune-related regulation as they develop; “DNA replication” and “mismatch repair” pathways are significantly enriched in CAFs at late developmental stages (cluster 1), which reflects that CAFs gradually play a regulatory role. These more complex and advanced roles of transcription can be seen.

CAFs are classified into 3 subtypes, named type1 cells, type2 cells, and type3 cells. C7 (Complement C7) and CNN1 are marker genes for type1 cells, and some studies have manifested that C7 can be used as a marker gene for immune CAFs [31]. However, other marker genes of immune CAFs, such as C3, IGF1, IL6, CXCL12, and other genes are not specifically expressed [31, 32]. Therefore, they cannot be defined as immune CAFs yet; CNN1, as a protein-coding gene, is mainly involved in the negative regulation of vascular smooth muscle cell proliferation [33, 34]. SELENOM and RACK are marker genes in type2 cells, and SELENOM exerts a great influence on the regulation of body weight and energy metabolism [35, 36]. CTGF and TNXB are marker genes for type 3 cells. CTGF plays a crucial part in chondrocyte proliferation and differentiation, such as cell adhesion, and is relevant to a platelet-derived growth factor [37, 38]. Based on the above analysis, it can be speculated that type1 cells are likely to be immune-associated CAFs, type2 cells are likely to be vascular-associated CAFs, and type3 cells are likely to be stroma-associated CAFs, although this speculation needs to be confirmed by more cell numbers and functional experiments.

Further, the communication between CAFs and several others was analyzed, obtaining the ligand-receptor pairs between CAFs and T and B cells. The communication between CAFs and immune cells also potentially reveals the role of CAFs in the tumour microenvironment. COL1A1- CD44 and COL1A2- CD44, as important ligand-receptor pairs between CAFs and T cells, are important signaling pathways for communication between CAFs and T cells. In cervical cancer, CAFs and B cells communicate through MIF-(CD74+CXCR4) and MIF-(CD74+CD44). MK signaling pathways are important pathways that mediate tumor cell growth, migration, and angiogenesis, and MK can play an important role as a potential tumor marker in tumor detection, prognosis, and treatment [39]. In our study, CAFs were confirmed to play a key role in the MK signaling pathway, capable of communicating well with several other cells in the MK signaling pathway. Additionally, key transcription factors for type1, type2, and type3 cells were obtained, namely TCF21, ZC3H11A, and MYEF2. TCF21 is an important transcription factor in fibroblasts and plays an important role in the differentiation of fibroblasts [40]. TCF21 is also involved in the repression of DNA-binding transcriptional activity [41]. ZC3H11A is mainly involved in the translocation of mature transcripts to the cytoplasm, thus affecting transcriptional processes [42]. The RNA-binding protein, MYEF2, can influence cell differentiation through PARylation regulation [43]. The transcription factors of the three cell types were modularized in this study, and it was found that important transcription factors were clustered in Module 1, which were significantly enriched in “cancer-related pathways”, “transcriptional misregulation in cancer”, “viral oncogenesis” and “human papillomavirus infection”, consistent with our study of cervical cancer and its characterization by HPV infection. Among the four genes associated with cervical cancer prognosis in CAFs, NR2F2 -AS1 can influence cervical cancer progression by regulating the miR-4429/mbd1 axis [44] and miR-221-3p promotes angiogenesis by targeting THBS2 and influences cervical carcinogenesis [45].

In conclusion, our study contributed to better understanding the role of CAFs in cervical cancer, providing a theoretical basis for further targeted therapies against CAFs.

## CONCLUSIONS

A total of 9 cell types were identified in this research, encompassing T cells, epithelial cells, smooth muscle cells, CAFs, endothelial cells, macrophages, B cells, lymphocytes, and plasma cells. CAFs were further classified into three cell subtypes, type1 cells, type2 cells, and type3 cells. And key transcription factors for three cell types, TCF21, ZC3H11A, and MYEF2 were acquired, revealing the communication between CAFs and several other cell types, and the important role of CAFs in the MK signaling pathway. scRNA-seq technology not only helped to explore the tumor heterogeneity of cervical cancer more deeply but also gave us further insight into the biological functions of CAFs in cervical cancer. Nevertheless, it is still necessary to collect more specimens, obtain more cells, and verify the specific mechanisms of CAFs in cervical cancer through experiments.

## Supplementary Material

Supplementary File 1

Supplementary File 2

## References

[1] Sawaya GF, Smith-McCune K, Kuppermann M. Cervical Cancer Screening: More Choices in 2019. JAMA. 2019; 321:2018–9. 10.1001/jama.2019.4595 31135834 PMC6656358

[2] Schiffman M, Solomon D. Clinical practice. Cervical-cancer screening with human papillomavirus and cytologic cotesting. N Engl J Med. 2013; 369:2324–31. 10.1056/NEJMcp1210379 24328466

[3] Gaffney DK, Hashibe M, Kepka D, Maurer KA, Werner TL. Too many women are dying from cervix cancer: Problems and solutions. Gynecol Oncol. 2018; 151:547–54. 10.1016/j.ygyno.2018.10.004 30301561 PMC6281756

[4] Cui Y, Guo G. Immunomodulatory Function of the Tumor Suppressor p53 in Host Immune Response and the Tumor Microenvironment. Int J Mol Sci. 2016; 17:1942. 10.3390/ijms17111942 27869779 PMC5133937

[5] Albini A, Bruno A, Noonan DM, Mortara L. Contribution to Tumor Angiogenesis From Innate Immune Cells Within the Tumor Microenvironment: Implications for Immunotherapy. Front Immunol. 2018; 9:527. 10.3389/fimmu.2018.00527 29675018 PMC5895776

[6] Svensson V, Vento-Tormo R, Teichmann SA. Exponential scaling of single-cell RNA-seq in the past decade. Nat Protoc. 2018; 13:599–604. 10.1038/nprot.2017.149 29494575

[7] Liu Q, Yu B, Tian Y, Dan J, Luo Y, Wu X. P53 Mutant p53N236S Regulates Cancer-Associated Fibroblasts Properties Through Stat3 Pathway. Onco Targets Ther. 2020; 13:1355–63. 10.2147/OTT.S229065 32104002 PMC7027832

[8] Cao G, Yue J, Ruan Y, Han Y, Zhi Y, Lu J, Liu M, Xu X, Wang J, Gu Q, Wen X, Gao J, Zhang Q, et al. Single-cell dissection of cervical cancer reveals key subsets of the tumor immune microenvironment. EMBO J. 2023; 42:e110757. 10.15252/embj.2022110757 37427448 PMC10425846

[9] Dobin A, Davis CA, Schlesinger F, Drenkow J, Zaleski C, Jha S, Batut P, Chaisson M, Gingeras TR. STAR: ultrafast universal RNA-seq aligner. Bioinformatics. 2013; 29:15–21. 10.1093/bioinformatics/bts635 23104886 PMC3530905

[10] Butler A, Hoffman P, Smibert P, Papalexi E, Satija R. Integrating single-cell transcriptomic data across different conditions, technologies, and species. Nat Biotechnol. 2018; 36:411–20. 10.1038/nbt.4096 29608179 PMC6700744

[11] McGinnis CS, Murrow LM, Gartner ZJ. DoubletFinder: Doublet Detection in Single-Cell RNA Sequencing Data Using Artificial Nearest Neighbors. Cell Syst. 2019; 8:329–37.e4. 10.1016/j.cels.2019.03.003 30954475 PMC6853612

[12] Korsunsky I, Millard N, Fan J, Slowikowski K, Zhang F, Wei K, Baglaenko Y, Brenner M, Loh PR, Raychaudhuri S. Fast, sensitive and accurate integration of single-cell data with Harmony. Nat Methods. 2019; 16:1289–96. 10.1038/s41592-019-0619-0 31740819 PMC6884693

[13] Zhou B, Jin W. Visualization of Single Cell RNA-Seq Data Using t-SNE in R. Methods Mol Biol. 2020; 2117:159–67. 10.1007/978-1-0716-0301-7_8 31960377

[14] Stuart T, Satija R. Integrative single-cell analysis. Nat Rev Genet. 2019; 20:257–72. 10.1038/s41576-019-0093-7 30696980

[15] Kang HM, Subramaniam M, Targ S, Nguyen M, Maliskova L, McCarthy E, Wan E, Wong S, Byrnes L, Lanata CM, Gate RE, Mostafavi S, Marson A, et al. Multiplexed droplet single-cell RNA-sequencing using natural genetic variation. Nat Biotechnol. 2018; 36:89–94. 10.1038/nbt.4042 29227470 PMC5784859

[16] Chen K, Liu X, Liu W, Wang F, Tian X, Yang Y. Development and validation of prognostic and diagnostic model for pancreatic ductal adenocarcinoma based on scRNA-seq and bulk-seq datasets. Hum Mol Genet. 2022; 31:1705–19. 10.1093/hmg/ddab343 34957503 PMC9122644

[17] Jin S, Guerrero-Juarez CF, Zhang L, Chang I, Ramos R, Kuan CH, Myung P, Plikus MV, Nie Q. Inference and analysis of cell-cell communication using CellChat. Nat Commun. 2021; 12:1088. 10.1038/s41467-021-21246-9 33597522 PMC7889871

[18] Matsui I, Matsumoto A, Inoue K, Katsuma Y, Yasuda S, Shimada K, Sakaguchi Y, Mizui M, Kaimori JY, Takabatake Y, Isaka Y. Single cell RNA sequencing uncovers cellular developmental sequences and novel potential intercellular communications in embryonic kidney. Sci Rep. 2021; 11:73. 10.1038/s41598-020-80154-y 33420268 PMC7794461

[19] Van de Sande B, Flerin C, Davie K, De Waegeneer M, Hulselmans G, Aibar S, Seurinck R, Saelens W, Cannoodt R, Rouchon Q, Verbeiren T, De Maeyer D, Reumers J, et al. A scalable SCENIC workflow for single-cell gene regulatory network analysis. Nat Protoc. 2020; 15:2247–76. 10.1038/s41596-020-0336-2 32561888

[20] Aibar S, González-Blas CB, Moerman T, Huynh-Thu VA, Imrichova H, Hulselmans G, Rambow F, Marine JC, Geurts P, Aerts J, van den Oord J, Atak ZK, Wouters J, Aerts S. SCENIC: single-cell regulatory network inference and clustering. Nat Methods. 2017; 14:1083–6. 10.1038/nmeth.4463 28991892 PMC5937676

[21] Suo S, Zhu Q, Saadatpour A, Fei L, Guo G, Yuan GC. Revealing the Critical Regulators of Cell Identity in the Mouse Cell Atlas. Cell Rep. 2018; 25:1436–45.e3. 10.1016/j.celrep.2018.10.045 30404000 PMC6281296

[22] Turajlic S, Sottoriva A, Graham T, Swanton C. Resolving genetic heterogeneity in cancer. Nat Rev Genet. 2019; 20:404–16. 10.1038/s41576-019-0114-6 30918367

[23] Cabili MN, Trapnell C, Goff L, Koziol M, Tazon-Vega B, Regev A, Rinn JL. Integrative annotation of human large intergenic noncoding RNAs reveals global properties and specific subclasses. Genes Dev. 2011; 25:1915–27. 10.1101/gad.17446611 21890647 PMC3185964

[24] Zhu Q, Wong AK, Krishnan A, Aure MR, Tadych A, Zhang R, Corney DC, Greene CS, Bongo LA, Kristensen VN, Charikar M, Li K, Troyanskaya OG. Targeted exploration and analysis of large cross-platform human transcriptomic compendia. Nat Methods. 2015; 12:211–4. 10.1038/nmeth.3249 25581801 PMC4768301

[25] Qiao N, Huang Y, Naveed H, Green CD, Han JD. CoCiter: an efficient tool to infer gene function by assessing the significance of literature co-citation. PLoS One. 2013; 8:e74074. 10.1371/journal.pone.0074074 24086311 PMC3781068

[26] Fuxman Bass JI, Diallo A, Nelson J, Soto JM, Myers CL, Walhout AJ. Using networks to measure similarity between genes: association index selection. Nat Methods. 2013; 10:1169–76. 10.1038/nmeth.2728 24296474 PMC3959882

[27] Kanzaki R, Pietras K. Heterogeneity of cancer-associated fibroblasts: Opportunities for precision medicine. Cancer Sci. 2020; 111:2708–17. 10.1111/cas.14537 32573845 PMC7419037

[28] Clevers H. Wnt/beta-catenin signaling in development and disease. Cell. 2006; 127:469–80. 10.1016/j.cell.2006.10.018 17081971

[29] McCoy MK, Tansey MG. TNF signaling inhibition in the CNS: implications for normal brain function and neurodegenerative disease. J Neuroinflammation. 2008; 5:45. 10.1186/1742-2094-5-45 18925972 PMC2577641

[30] Amatya N, Garg AV, Gaffen SL. IL-17 Signaling: The Yin and the Yang. Trends Immunol. 2017; 38:310–22. 10.1016/j.it.2017.01.006 28254169 PMC5411326

[31] Zhang M, Yang H, Wan L, Wang Z, Wang H, Ge C, Liu Y, Hao Y, Zhang D, Shi G, Gong Y, Ni Y, Wang C, et al. Single-cell transcriptomic architecture and intercellular crosstalk of human intrahepatic cholangiocarcinoma. J Hepatol. 2020; 73:1118–30. 10.1016/j.jhep.2020.05.039 32505533

[32] Chen Z, Zhou L, Liu L, Hou Y, Xiong M, Yang Y, Hu J, Chen K. Single-cell RNA sequencing highlights the role of inflammatory cancer-associated fibroblasts in bladder urothelial carcinoma. Nat Commun. 2020; 11:5077. 10.1038/s41467-020-18916-5 33033240 PMC7545162

[33] Norman TM, Horlbeck MA, Replogle JM, Ge AY, Xu A, Jost M, Gilbert LA, Weissman JS. Exploring genetic interaction manifolds constructed from rich single-cell phenotypes. Science. 2019; 365:786–93. 10.1126/science.aax4438 31395745 PMC6746554

[34] Bock LJ, Pagliuca C, Kobayashi N, Grove RA, Oku Y, Shrestha K, Alfieri C, Golfieri C, Oldani A, Dal Maschio M, Bermejo R, Hazbun TR, Tanaka TU, De Wulf P. Cnn1 inhibits the interactions between the KMN complexes of the yeast kinetochore. Nat Cell Biol. 2012; 14:614–24. 10.1038/ncb2495 22561345 PMC3438452

[35] Gong T, Hashimoto AC, Sasuclark AR, Khadka VS, Gurary A, Pitts MW. Selenoprotein M Promotes Hypothalamic Leptin Signaling and Thioredoxin Antioxidant Activity. Antioxid Redox Signal. 2021; 35:775–87. 10.1089/ars.2018.7594 30648404 PMC8617589

[36] Choi Y, Choi H, Lee AC, Lee H, Kwon S. A Reconfigurable DNA Accordion Rack. Angew Chem Int Ed Engl. 2018; 57:2811–5. 10.1002/anie.201709362 29368437

[37] Makino Y, Hikita H, Kodama T, Shigekawa M, Yamada R, Sakamori R, Eguchi H, Morii E, Yokoi H, Mukoyama M, Hiroshi S, Tatsumi T, Takehara T. CTGF Mediates Tumor-Stroma Interactions between Hepatoma Cells and Hepatic Stellate Cells to Accelerate HCC Progression. Cancer Res. 2018; 78:4902–14. 10.1158/0008-5472.CAN-17-3844 29967264

[38] Gbadegesin RA, Brophy PD, Adeyemo A, Hall G, Gupta IR, Hains D, Bartkowiak B, Rabinovich CE, Chandrasekharappa S, Homstad A, Westreich K, Wu G, Liu Y, et al. TNXB mutations can cause vesicoureteral reflux. J Am Soc Nephrol. 2013; 24:1313–22. 10.1681/ASN.2012121148 23620400 PMC3736717

[39] Filippou PS, Karagiannis GS, Constantinidou A. Midkine (MDK) growth factor: a key player in cancer progression and a promising therapeutic target. Oncogene. 2020; 39:2040–54. 10.1038/s41388-019-1124-8 31801970

[40] Kanisicak O, Khalil H, Ivey MJ, Karch J, Maliken BD, Correll RN, Brody MJ, J Lin SC, Aronow BJ, Tallquist MD, Molkentin JD. Genetic lineage tracing defines myofibroblast origin and function in the injured heart. Nat Commun. 2016; 7:12260. 10.1038/ncomms12260 27447449 PMC5512625

[41] Xie Y, Martin KA. TCF21: Flipping the Phenotypic Switch in SMC. Circ Res. 2020; 126:530–2. 10.1161/CIRCRESAHA.120.316533 32078454 PMC7041869

[42] Younis S, Kamel W, Falkeborn T, Wang H, Yu D, Daniels R, Essand M, Hinkula J, Akusjärvi G, Andersson L. Multiple nuclear-replicating viruses require the stress-induced protein ZC3H11A for efficient growth. Proc Natl Acad Sci USA. 2018; 115:E3808–16. 10.1073/pnas.1722333115 29610341 PMC5910864

[43] Wang Y, Zhang Y, Zhang S, Kim B, Hull VL, Xu J, Prabhu P, Gregory M, Martinez-Cerdeno V, Zhan X, Deng W, Guo F. PARP1-mediated PARylation activity is essential for oligodendroglial differentiation and CNS myelination. Cell Rep. 2021; 37:109695. 10.1016/j.celrep.2021.109695 34610310 PMC9586836

[44] Liu D, Huang K, Wang T, Zhang X, Liu W, Yue X, Wu J. NR2F2-AS1 accelerates cell proliferation through regulating miR-4429/MBD1 axis in cervical cancer. Biosci Rep. 2020; 40:BSR20194282. 10.1042/BSR20194282 32469064 PMC7295628

[45] Wu XG, Zhou CF, Zhang YM, Yan RM, Wei WF, Chen XJ, Yi HY, Liang LJ, Fan LS, Liang L, Wu S, Wang W. Cancer-derived exosomal miR-221-3p promotes angiogenesis by targeting THBS2 in cervical squamous cell carcinoma. Angiogenesis. 2019; 22:397–410. 10.1007/s10456-019-09665-1 30993566

