# Supplementary material for: Analysis of cancer-associated fibroblasts in cervical cancer by single-cell RNA sequencing
Source: Aging (Albany NY). 2023 Dec 28;15(24):15340–59. doi: 10.18632/aging.205353 (PMC10781451; doi:10.18632/aging.205353)
Supplement: Supplementary File 2 [file aging-15-205353-s002.docx]

**Supplementary File 2. The whole code script in this study.**

rm(list = ls())

#install.packages("Seurat")

####逐个处理单细胞数据

library(Seurat)##version 4.0.5

library(dplyr)

library(future)

library(future.apply)

library(DoubletFinder)

plan("multiprocess", workers = 8) ###compute cores

options(future.globals.maxSize = 8000 * 1024^2)

getwd()

setwd("E:\\宫颈癌单细胞\\单细胞\\1、原始数据处理")

list.files(path = "./",pattern = "^CC")

####SRR781####

SRR781<-Read10X("./CC4/")

SRR781[1:5,1:5]

SRR781_object<- CreateSeuratObject(counts = SRR781, project = "SRR781", min.cells = 0, min.features = 200)

SRR781_object[["percent.mt"]] <- PercentageFeatureSet(SRR781_object, pattern = "^MT-")

hist(SRR781_object[["percent.mt"]]$percent.mt)

VlnPlot(SRR781_object, features = c("nFeature_RNA", "nCount_RNA", "percent.mt"), ncol = 3)

plot1 <- FeatureScatter(SRR781_object, feature1 = "nCount_RNA", feature2 = "percent.mt")

plot2 <- FeatureScatter(SRR781_object, feature1 = "nCount_RNA", feature2 = "nFeature_RNA")

CombinePlots(plots = list(plot1,plot2))

SRR781_object

##select the cells with 300 genes at least and 4000 at most, the percent of mitochondrion genes is less than 10%

SRR781_val<- subset(SRR781_object, subset = nFeature_RNA > 200 & nFeature_RNA < 7000 & percent.mt < 20)

SRR781_val@meta.data[1:5,]

colname<-paste("CC4_",colnames(SRR781_val),sep="")

colname

SRR781_val<-RenameCells(object = SRR781_val,colname)

#rm(list = c("BC2","bc2_object"))

SRR781_val@meta.data[1:5,]

SRR781_val$tissue_type<-"Normal"

SRR781_val <- NormalizeData(SRR781_val, normalization.method = "LogNormalize", scale.factor = 10000)

SRR781_val <- FindVariableFeatures(SRR781_val, selection.method = "vst", nfeatures = 2000)

###scaling the data###

all.genes <- rownames(SRR781_val)

SRR781_val <- ScaleData(SRR781_val,vars.to.regress = c("percent.mt"))

SRR781_val

###perform linear dimensional reduction###

SRR781_val <- RunPCA(SRR781_val, features = VariableFeatures(object = SRR781_val))

print(SRR781_val[["pca"]], dims = 1:5, nfeatures = 5)

#dev.off()

VizDimLoadings(SRR781_val, dims = 1:2, reduction = "pca")

ElbowPlot(SRR781_val,ndims = 50)

####cluster the cells###

SRR781_val <- FindNeighbors(SRR781_val, dims = 1:30)

####Run non-linear dimensional reduction (UMAP/tSNE)###

SRR781_val <- RunUMAP(SRR781_val, dims = 1:30)

SRR781_val<-RunTSNE(SRR781_val,dims=1:30)

SRR781_val <- FindClusters(SRR781_val, resolution = 0.6)

DimPlot(SRR781_val, reduction = "umap",label = T)

#?DimPlot

DimPlot(SRR781_val, reduction = "tsne",label = T)

SRR781.markers <- FindAllMarkers(SRR781_val, only.pos = TRUE, min.pct = 0.25, logfc.threshold = 0.25)

write.csv(SRR781.markers,"./CC4/CC4.markers.csv")

#marker <- FindMarkers(SRR781_val, ident.1 = "3",ident.2 = "7",only.pos = TRUE, min.pct = 0.25, logfc.threshold = 0.25)

######biomarkers###

###epithelial cell

FeaturePlot(SRR781_val,features = c("EPCAM","CDH1","KRT7","KRT5","UPK1A","KRT20"),reduction = "tsne",label = T)

##TNK cell

FeaturePlot(SRR781_val,features = c("CD3D","CD8A","CD4","NCAM1"),reduction = "tsne",label = T)

##endothelial cell

FeaturePlot(SRR781_val,features = c("VWF","PECAM1"),reduction = "tsne",label = T)

####fibroblasts and pericytes

FeaturePlot(SRR781_val,features = c("RGS5","COL1A1","PDGFRA","PDGFRB","DES"),reduction = "tsne",label = T)

###B cell and plasma

FeaturePlot(SRR781_val,features = c("CD19","JCHAIN","CD79A"),reduction = "tsne",label = T)

###myeloid cell

FeaturePlot(SRR781_val,features = c("CD74","CD14","FCGR3A","S100A8"),reduction = "tsne",label = T)

###mast cell

FeaturePlot(SRR781_val,features = c("CPA3"),reduction = "tsne",label = T)

Idents(SRR781_val)<-SRR781_val$RNA_snn_res.0.6

SRR781_val<-RenameIdents(SRR781_val,"5"="Epithelial","21"="Epithelial","16"="Epithelial","1"="Fibroblast","22"="Fibroblast","23"="Fibroblast","4"="Pericytes","8"="Pericytes","25"="Pericytes",

"14"="MAST","10"="B",

"11"="Plasma_B","6"="T/NK","3"="T/NK","12"="T/NK","19"="T/NK","9"="Myeloid","17"="Myeloid","18"="Myeloid","0"="Endothelial","2"="Endothelial","7"="Endothelial","13"="Endothelial","15"="Endothelial","24"="Endothelial","20"="Neuron")

DimPlot(SRR781_val,reduction = "tsne",label = T)

DimPlot(SRR781_val,reduction = "umap",label = T)

table(Idents(SRR781_val))###查看有多少个细胞

SRR781_val$cell_cluster_origin<-Idents(SRR781_val)

SRR781_val@meta.data[1:5,]

#save.image(file="./CC4/CC4.RData")

#load("./SRR781_outs/SRR781_val.RData")

SRR781_val$cluster<-Idents(SRR781_val)

saveRDS(SRR781_val,file = "./CC4/CC4_val.rds")

SRR781_val<-readRDS("./SRR81_/SRR781_val.rds")

####auto cellular annotation#

#install.packages("shinythemes")

#devtools::install_github("ggjlab/scHCL")###郭国冀老师团队

library(scHCL)

hcl_result <- scHCL(scdata = SRR781_val@assays$RNA@data, numbers_plot = 1)###time comsuming take several mins，上课时不要运行这个，需要很长时间，建议空闲时跑

cellassign<-table(Idents(SRR781_val),hcl_result$scHCL_probility$`Cell type`)

cellassign

cellassign<-table(SRR781_val$RNA_snn_res.0.6,hcl_result$scHCL_probility$`Cell type`)

cellassign

library(stringr)

cell_predict<-data.frame(hcl_result$scHCL_probility)

table(rownames(SRR781_val@meta.data)==cell_predict$Cell)

cell_predict$cell_predict<-str_split(cell_predict$Cell.type,"[.]",simplify = T)[,1]

table(SRR781_val$RNA_snn_res.0.6,cell_predict$cell_predict)

table(SRR781_val$cluster,cell_predict$cell_predict)

SRR781_val$cell_predict<-cell_predict$cell_predict

SRR781_val@meta.data[1:3,]

####Doublets identification#

library(DoubletFinder)

###完整的分析：前面和建立单细胞Seurat对象一样，因为前面跑过了，这里都省略

if (TRUE){

SRR781<-Read10X(data.dir = "SRR80_outs/filtered_feature_bc_matrix/")

SRR781[1:5,1:5]

SRR781_object<- CreateSeuratObject(counts = SRR781, project = "SRR781", min.cells = 0, min.features = 200)

SRR781_object[["percent.mt"]] <- PercentageFeatureSet(SRR781_object, pattern = "^MT-")

hist(SRR781_object[["percent.mt"]]$percent.mt)

VlnPlot(SRR781_object, features = c("nFeature_RNA", "nCount_RNA", "percent.mt"), ncol = 3)

plot1 <- FeatureScatter(SRR781_object, feature1 = "nCount_RNA", feature2 = "percent.mt")

plot2 <- FeatureScatter(SRR781_object, feature1 = "nCount_RNA", feature2 = "nFeature_RNA")

CombinePlots(plots = list(plot1,plot2))

SRR781_object

##select the cells with 300 genes at least and 4000 at most, the percent of mitochondrion genes is less than 10%

SRR781_val<- subset(SRR781_object, subset = nFeature_RNA > 200 & nFeature_RNA < 7000 & percent.mt < 20)

SRR781_val@meta.data[1:5,]

colname<-paste("SRR781_",colnames(SRR781_val),sep="")

colname

SRR781_val<-RenameCells(object = SRR781_val,colname)

#rm(list = c("BC2","bc2_object"))

SRR781_val@meta.data[1:5,]

SRR781_val$tissue_type<-"Normal"

SRR781_val <- NormalizeData(SRR781_val, normalization.method = "LogNormalize", scale.factor = 10000)

SRR781_val <- FindVariableFeatures(SRR781_val, selection.method = "vst", nfeatures = 2000)

###scaling the data###

all.genes <- rownames(SRR781_val)

SRR781_val <- ScaleData(SRR781_val,vars.to.regress = c("percent.mt"))

SRR781_val

###perform linear dimensional reduction###

SRR781_val <- RunPCA(SRR781_val, features = VariableFeatures(object = SRR781_val))

print(SRR781_val[["pca"]], dims = 1:5, nfeatures = 5)

#dev.off()

VizDimLoadings(SRR781_val, dims = 1:2, reduction = "pca")

ElbowPlot(SRR781_val,ndims = 50)

####cluster the cells###

SRR781_val <- FindNeighbors(SRR781_val, dims = 1:30)

####Run non-linear dimensional reduction (UMAP/tSNE)###

SRR781_val <- RunUMAP(SRR781_val, dims = 1:30)

SRR781_val<-RunTSNE(SRR781_val,dims=1:30)

SRR781_val <- FindClusters(SRR781_val, resolution = 0.6)

}

SRR781_val@meta.data[1:5,]

####因为前面跑过，所以直接从这步开始计算###

SRR781_val_db<-SRR781_val

sweep.res.list_SRR781_val <- paramSweep_v3(SRR781_val_db, PCs = 1:30)

sweep.stats_SRR781_val <- summarizeSweep(sweep.res.list_SRR781_val, GT = FALSE)

bcmvn_SRR781_val<-find.pK(sweep.stats_SRR781_val)

pK_value <- as.numeric(as.character(bcmvn_SRR781_val$pK[bcmvn_SRR781_val$BCmetric == max(bcmvn_SRR781_val$BCmetric)]))

annotations <- SRR781_val_db@meta.data$RNA_snn_res.0.6

homotypic.prop <- modelHomotypic(annotations) ####get the estimated number

nExp_poi <- round(homotypic.prop*length(SRR781_val_db@meta.data$orig.ident))

nExp_poi.adj <- round(nExp_poi*(1-homotypic.prop))

pN_value <- 0.25

pANN_value <- paste0("pANN_",pN_value,"_",pK_value,'_',nExp_poi)

SRR781_val_db <- doubletFinder_v3(SRR781_val_db, PCs = 1:30, pN = pN_value, pK = pK_value, nExp = nExp_poi, reuse.pANN = FALSE)

SRR781_val_db <- doubletFinder(SRR781_val_db, PCs = 1:30, pN = pN_value, pK = pK_value, nExp = nExp_poi.adj, reuse.pANN = pANN_value)

SRR781_val_db@meta.data[1:5,]

SRR781_val$doublets<-SRR781_val_db$DF.classifications_0.25_0.2_497

SRR781_val@meta.data[1:5,]

####saveRDS###

saveRDS(SRR781_val,file = "./SRR81_outs/SRR781_val.rds")

DimPlot(SRR781_val,reduction = "tsne",group.by = "doublets")

####SRR782####

rm(list = ls())

SRR782<-Read10X(data.dir = "./CC1/")

SRR782[1:5,1:5]

SRR782_object<- CreateSeuratObject(counts = SRR782, project = "SRR782", min.cells = 0, min.features = 200)

SRR782_object[["percent.mt"]] <- PercentageFeatureSet(SRR782_object, pattern = "^MT-")

hist(SRR782_object[["percent.mt"]]$percent.mt)

VlnPlot(SRR782_object, features = c("nFeature_RNA", "nCount_RNA", "percent.mt"), ncol = 3)

plot1 <- FeatureScatter(SRR782_object, feature1 = "nCount_RNA", feature2 = "percent.mt")

plot2 <- FeatureScatter(SRR782_object, feature1 = "nCount_RNA", feature2 = "nFeature_RNA")

CombinePlots(plots = list(plot1,plot2))

SRR782_object

##select the cells with 300 genes at least and 4000 at most, the percent of mitochondrion genes is less than 10%

SRR782_val<- subset(SRR782_object, subset = nFeature_RNA > 200 & nFeature_RNA < 7000 & percent.mt < 20)

SRR782_val@meta.data[1:5,]

colname<-paste("CC1",colnames(SRR782_val),sep="")

colname

SRR782_val<-RenameCells(object = SRR782_val,colname)

#rm(list = c("BC2","bc2_object"))

SRR782_val@meta.data[1:5,]

SRR782_val$tissue_type<-"Tumor"

SRR782_val <- NormalizeData(SRR782_val, normalization.method = "LogNormalize", scale.factor = 10000)

SRR782_val <- FindVariableFeatures(SRR782_val, selection.method = "vst", nfeatures = 2000)

###scaling the data###

all.genes <- rownames(SRR782_val)

SRR782_val <- ScaleData(SRR782_val,vars.to.regress = c("percent.mt"))

SRR782_val

###perform linear dimensional reduction###

SRR782_val <- RunPCA(SRR782_val, features = VariableFeatures(object = SRR782_val))

print(SRR782_val[["pca"]], dims = 1:5, nfeatures = 5)

#dev.off()

VizDimLoadings(SRR782_val, dims = 1:2, reduction = "pca")

ElbowPlot(SRR782_val,ndims = 50)

####cluster the cells###

SRR782_val <- FindNeighbors(SRR782_val, dims = 1:30)

####Run non-linear dimensional reduction (UMAP/tSNE)###

SRR782_val <- RunUMAP(SRR782_val, dims = 1:30)

SRR782_val<-RunTSNE(SRR782_val,dims=1:30)

SRR782_val <- FindClusters(SRR782_val, resolution = 0.8)

DimPlot(SRR782_val, reduction = "umap",label = T)

?DimPlot

DimPlot(SRR782_val, reduction = "tsne",label = T)

SRR782.markers <- FindAllMarkers(SRR782_val, only.pos = TRUE, min.pct = 0.25, logfc.threshold = 0.25)

write.csv(SRR782.markers,"./SRR82_outs/SRR782.markers.csv")

#marker <- FindMarkers(SRR782_val, ident.1 = "3",ident.2 = "7",only.pos = TRUE, min.pct = 0.25, logfc.threshold = 0.25)

######biomarkers###

###epithelial cell

FeaturePlot(SRR782_val,features = c("EPCAM","CDH1","KRT7","KRT5","UPK1A","KRT20"),reduction = "tsne",label = T)

##TNK cell

FeaturePlot(SRR782_val,features = c("CD3D","CD8A","CD4","NCAM1"),reduction = "tsne",label = T)

##endothelial cell

FeaturePlot(SRR782_val,features = c("VWF","PECAM1"),reduction = "tsne",label = T)

####fibroblasts and pericytes

FeaturePlot(SRR782_val,features = c("RGS5","COL1A1","PDGFRA","PDGFRB","DES"),reduction = "tsne",label = T)

###B cell and plasma

FeaturePlot(SRR782_val,features = c("CD19","JCHAIN","CD79A"),reduction = "tsne",label = T)

###myeloid cell

FeaturePlot(SRR782_val,features = c("CD74","CD14","FCGR3A","S100A8"),reduction = "tsne",label = T)

###mast cell

FeaturePlot(SRR782_val,features = c("CPA3"),reduction = "tsne",label = T)

Idents(SRR782_val)<-SRR782_val$RNA_snn_res.0.6

SRR782_val<-RenameIdents(SRR782_val,"0"="Epithelial","1"="Epithelial","2"="Epithelial","3"="Epithelial","4"="Epithelial","5"="Epithelial","9"="Epithelial","10"="Epithelial",

"8"="Fibroblast","6"="T/NK","7"="Myeloid")

DimPlot(SRR782_val,reduction = "tsne",label = T)

DimPlot(SRR782_val,reduction = "umap",label = T)

table(Idents(SRR782_val))###查看有多少个细胞

SRR782_val$cell_cluster_origin<-Idents(SRR782_val)

SRR782_val@meta.data[1:5,]

saveRDS(SRR782_val,file = "./SRR82_outs/SRR782_val.rds")

#save.image(file="./SRR782_outs/SRR782_val.RData")

#load("./SRR782_outs/SRR782_val.RData")

SRR782_val$cluster<-Idents(SRR782_val)

####auto cellular annotation#

#install.packages("shinythemes")

#devtools::install_github("ggjlab/scHCL")###郭国冀老师团队

library(scHCL)

hcl_result <- scHCL(scdata = SRR782_val@assays$RNA@data, numbers_plot = 1)###time comsuming take several mins

cellassign<-table(Idents(SRR782_val),hcl_result$scHCL_probility$`Cell type`)

cellassign

cellassign<-table(SRR782_val$RNA_snn_res.0.6,hcl_result$scHCL_probility$`Cell type`)

cellassign

library(stringr)

cell_predict<-data.frame(hcl_result$scHCL_probility)

table(rownames(SRR782_val@meta.data)==cell_predict$Cell)

cell_predict$cell_predict<-str_split(cell_predict$Cell.type,"[.]",simplify = T)[,1]

table(SRR782_val$RNA_snn_res.0.6,cell_predict$cell_predict)

table(SRR782_val$cluster,cell_predict$cell_predict)

SRR782_val$cell_predict<-cell_predict$cell_predict

SRR782_val@meta.data[1:3,]

####Doublets identification#

library(DoubletFinder)

###完整的分析：前面和建立单细胞Seurat对象一样，因为前面跑过了，这里都省略

if (TRUE){

SRR782<-Read10X(data.dir = "SRR80_outs//filtered_feature_bc_matrix/")

SRR782[1:5,1:5]

SRR782_object<- CreateSeuratObject(counts = SRR782, project = "SRR782", min.cells = 0, min.features = 200)

SRR782_object[["percent.mt"]] <- PercentageFeatureSet(SRR782_object, pattern = "^MT-")

hist(SRR782_object[["percent.mt"]]$percent.mt)

VlnPlot(SRR782_object, features = c("nFeature_RNA", "nCount_RNA", "percent.mt"), ncol = 3)

plot1 <- FeatureScatter(SRR782_object, feature1 = "nCount_RNA", feature2 = "percent.mt")

plot2 <- FeatureScatter(SRR782_object, feature1 = "nCount_RNA", feature2 = "nFeature_RNA")

CombinePlots(plots = list(plot1,plot2))

SRR782_object

##select the cells with 300 genes at least and 4000 at most, the percent of mitochondrion genes is less than 10%

SRR782_val<- subset(SRR782_object, subset = nFeature_RNA > 200 & nFeature_RNA < 7000 & percent.mt < 20)

SRR782_val@meta.data[1:5,]

colname<-paste("SRR782_",colnames(SRR782_val),sep="")

colname

SRR782_val<-RenameCells(object = SRR782_val,colname)

#rm(list = c("BC2","bc2_object"))

SRR782_val@meta.data[1:5,]

SRR782_val$tissue_type<-"Normal"

SRR782_val <- NormalizeData(SRR782_val, normalization.method = "LogNormalize", scale.factor = 10000)

SRR782_val <- FindVariableFeatures(SRR782_val, selection.method = "vst", nfeatures = 2000)

###scaling the data###

all.genes <- rownames(SRR782_val)

SRR782_val <- ScaleData(SRR782_val,vars.to.regress = c("percent.mt"))

SRR782_val

###perform linear dimensional reduction###

SRR782_val <- RunPCA(SRR782_val, features = VariableFeatures(object = SRR782_val))

print(SRR782_val[["pca"]], dims = 1:5, nfeatures = 5)

#dev.off()

VizDimLoadings(SRR782_val, dims = 1:2, reduction = "pca")

ElbowPlot(SRR782_val,ndims = 50)

####cluster the cells###

SRR782_val <- FindNeighbors(SRR782_val, dims = 1:30)

####Run non-linear dimensional reduction (UMAP/tSNE)###

SRR782_val <- RunUMAP(SRR782_val, dims = 1:30)

SRR782_val<-RunTSNE(SRR782_val,dims=1:30)

SRR782_val <- FindClusters(SRR782_val, resolution = 0.6)

}

SRR782_val@meta.data[1:5,]

####因为前面跑过，所以直接从这步开始计算###

SRR782_val_db<-SRR782_val

sweep.res.list_SRR782_val <- paramSweep_v3(SRR782_val_db, PCs = 1:30)

sweep.stats_SRR782_val <- summarizeSweep(sweep.res.list_SRR782_val, GT = FALSE)

bcmvn_SRR782_val<-find.pK(sweep.stats_SRR782_val)

pK_value <- as.numeric(as.character(bcmvn_SRR782_val$pK[bcmvn_SRR782_val$BCmetric == max(bcmvn_SRR782_val$BCmetric)]))

annotations <- SRR782_val_db@meta.data$RNA_snn_res.0.6

homotypic.prop <- modelHomotypic(annotations) ####get the estimated number

nExp_poi <- round(homotypic.prop*length(SRR782_val_db@meta.data$orig.ident))

nExp_poi.adj <- round(nExp_poi*(1-homotypic.prop))

pN_value <- 0.25

pANN_value <- paste0("pANN_",pN_value,"_",pK_value,'_',nExp_poi)

SRR782_val_db <- doubletFinder_v3(SRR782_val_db, PCs = 1:30, pN = pN_value, pK = pK_value, nExp = nExp_poi, reuse.pANN = FALSE)

SRR782_val_db <- doubletFinder(SRR782_val_db, PCs = 1:30, pN = pN_value, pK = pK_value, nExp = nExp_poi.adj, reuse.pANN = pANN_value)

SRR782_val_db@meta.data[1:5,]

SRR782_val$doublets<-SRR782_val_db$DF.classifications_0.25_0.06_181

SRR782_val@meta.data[1:5,]

####saveRDS###

saveRDS(SRR782_val,file = "./SRR82_outs/SRR782_val.rds")

DimPlot(SRR782_val,reduction = "tsne",group.by = "doublets")

####SRR783####

rm(list = ls())

SRR783<-Read10X(data.dir = "SRR83_outs/filtered_feature_bc_matrix/")

SRR783[1:5,1:5]

SRR783_object<- CreateSeuratObject(counts = SRR783, project = "SRR783", min.cells = 0, min.features = 200)

SRR783_object[["percent.mt"]] <- PercentageFeatureSet(SRR783_object, pattern = "^MT-")

hist(SRR783_object[["percent.mt"]]$percent.mt)

VlnPlot(SRR783_object, features = c("nFeature_RNA", "nCount_RNA", "percent.mt"), ncol = 3)

plot1 <- FeatureScatter(SRR783_object, feature1 = "nCount_RNA", feature2 = "percent.mt")

plot2 <- FeatureScatter(SRR783_object, feature1 = "nCount_RNA", feature2 = "nFeature_RNA")

CombinePlots(plots = list(plot1,plot2))

SRR783_object

##select the cells with 300 genes at least and 4000 at most, the percent of mitochondrion genes is less than 10%

SRR783_val<- subset(SRR783_object, subset = nFeature_RNA > 200 & nFeature_RNA < 7000 & percent.mt < 20)

SRR783_val@meta.data[1:5,]

colname<-paste("SRR783_",colnames(SRR783_val),sep="")

colname

SRR783_val<-RenameCells(object = SRR783_val,colname)

#rm(list = c("BC2","bc2_object"))

SRR783_val@meta.data[1:5,]

SRR783_val$tissue_type<-"Tumor"

SRR783_val

SRR783_val <- NormalizeData(SRR783_val, normalization.method = "LogNormalize", scale.factor = 10000)

SRR783_val <- FindVariableFeatures(SRR783_val, selection.method = "vst", nfeatures = 2000)

###scaling the data###

all.genes <- rownames(SRR783_val)

SRR783_val <- ScaleData(SRR783_val,vars.to.regress = c("percent.mt"))

SRR783_val

###perform linear dimensional reduction###

SRR783_val <- RunPCA(SRR783_val, features = VariableFeatures(object = SRR783_val))

print(SRR783_val[["pca"]], dims = 1:5, nfeatures = 5)

#dev.off()

VizDimLoadings(SRR783_val, dims = 1:2, reduction = "pca")

ElbowPlot(SRR783_val,ndims = 50)

####cluster the cells###

SRR783_val <- FindNeighbors(SRR783_val, dims = 1:30)

####Run non-linear dimensional reduction (UMAP/tSNE)###

SRR783_val <- RunUMAP(SRR783_val, dims = 1:30)

SRR783_val<-RunTSNE(SRR783_val,dims=1:30)

SRR783_val <- FindClusters(SRR783_val, resolution = 0.6)

DimPlot(SRR783_val, reduction = "umap",label = T)

?DimPlot

DimPlot(SRR783_val, reduction = "tsne",label = T)

SRR783.markers <- FindAllMarkers(SRR783_val, only.pos = TRUE, min.pct = 0.25, logfc.threshold = 0.25)

write.csv(SRR783.markers,"./SRR83_outs/SRR783.markers.csv")

#marker <- FindMarkers(SRR783_val, ident.1 = "3",ident.2 = "7",only.pos = TRUE, min.pct = 0.25, logfc.threshold = 0.25)

######biomarkers###

###epithelial cell

FeaturePlot(SRR783_val,features = c("EPCAM","CDH1","KRT7","KRT5","UPK1A","KRT20"),reduction = "tsne",label = T)

##TNK cell

FeaturePlot(SRR783_val,features = c("CD3D","CD8A","CD4","NCAM1"),reduction = "tsne",label = T)

##endothelial cell

FeaturePlot(SRR783_val,features = c("VWF","PECAM1"),reduction = "tsne",label = T)

####fibroblasts and pericytes

FeaturePlot(SRR783_val,features = c("RGS5","COL1A1","PDGFRA","PDGFRB","DES"),reduction = "tsne",label = T)

###B cell and plasma

FeaturePlot(SRR783_val,features = c("CD19","JCHAIN","CD79A"),reduction = "tsne",label = T)

###myeloid cell

FeaturePlot(SRR783_val,features = c("CD74","CD14","FCGR3A","S100A8"),reduction = "tsne",label = T)

###mast cell

FeaturePlot(SRR783_val,features = c("CPA3"),reduction = "tsne",label = T)

Idents(SRR783_val)<-SRR783_val$RNA_snn_res.0.6

SRR783_val<-RenameIdents(SRR783_val,"0"="Epithelial","1"="Epithelial","2"="Epithelial","3"="Epithelial","5"="Epithelial","6"="Epithelial","7"="Epithelial","12"="Epithelial",

"13"="Fibroblast","9"="Pericytes","14"="MAST","15"="B",

"4"="T/NK","10"="T/NK","8"="Myeloid","11"="Endothelial")

DimPlot(SRR783_val,reduction = "tsne",label = T)

DimPlot(SRR783_val,reduction = "umap",label = T)

table(Idents(SRR783_val))###查看有多少个细胞

SRR783_val$cell_cluster_origin<-Idents(SRR783_val)

SRR783_val@meta.data[1:5,]

saveRDS(SRR783_val,file = "./SRR83_outs/SRR783_val.rds")

#save.image(file="./SRR783_outs/SRR783_val.RData")

#load("./SRR783_outs/SRR783_val.RData")

SRR783_val$cluster<-Idents(SRR783_val)

SRR783_val<-SRR783_val[,1:3000]

####auto cellular annotation#

#install.packages("shinythemes")

#devtools::install_github("ggjlab/scHCL")###郭国冀老师团队

library(scHCL)

hcl_result <- scHCL(scdata = SRR783_val@assays$RNA@data, numbers_plot = 1)###time comsuming take several mins

cellassign<-table(Idents(SRR783_val),hcl_result$scHCL_probility$`Cell type`)

cellassign

cellassign<-table(SRR783_val$RNA_snn_res.0.6,hcl_result$scHCL_probility$`Cell type`)

cellassign

library(stringr)

cell_predict<-data.frame(hcl_result$scHCL_probility)

table(rownames(SRR783_val@meta.data)==cell_predict$Cell)

cell_predict$cell_predict<-str_split(cell_predict$Cell.type,"[.]",simplify = T)[,1]

table(SRR783_val$RNA_snn_res.0.6,cell_predict$cell_predict)

table(SRR783_val$cluster,cell_predict$cell_predict)

SRR783_val$cell_predict<-cell_predict$cell_predict

SRR783_val@meta.data[1:3,]

####Doublets identification#

library(DoubletFinder)

###完整的分析：前面和建立单细胞Seurat对象一样，因为前面跑过了，这里都省略

if (TRUE){

SRR783<-Read10X(data.dir = "SRR80_outs//filtered_feature_bc_matrix/")

SRR783[1:5,1:5]

SRR783_object<- CreateSeuratObject(counts = SRR783, project = "SRR783", min.cells = 0, min.features = 200)

SRR783_object[["percent.mt"]] <- PercentageFeatureSet(SRR783_object, pattern = "^MT-")

hist(SRR783_object[["percent.mt"]]$percent.mt)

VlnPlot(SRR783_object, features = c("nFeature_RNA", "nCount_RNA", "percent.mt"), ncol = 3)

plot1 <- FeatureScatter(SRR783_object, feature1 = "nCount_RNA", feature2 = "percent.mt")

plot2 <- FeatureScatter(SRR783_object, feature1 = "nCount_RNA", feature2 = "nFeature_RNA")

CombinePlots(plots = list(plot1,plot2))

SRR783_object

##select the cells with 300 genes at least and 4000 at most, the percent of mitochondrion genes is less than 10%

SRR783_val<- subset(SRR783_object, subset = nFeature_RNA > 200 & nFeature_RNA < 7000 & percent.mt < 20)

SRR783_val@meta.data[1:5,]

colname<-paste("SRR783_",colnames(SRR783_val),sep="")

colname

SRR783_val<-RenameCells(object = SRR783_val,colname)

#rm(list = c("BC2","bc2_object"))

SRR783_val@meta.data[1:5,]

SRR783_val$tissue_type<-"Normal"

SRR783_val <- NormalizeData(SRR783_val, normalization.method = "LogNormalize", scale.factor = 10000)

SRR783_val <- FindVariableFeatures(SRR783_val, selection.method = "vst", nfeatures = 2000)

###scaling the data###

all.genes <- rownames(SRR783_val)

SRR783_val <- ScaleData(SRR783_val,vars.to.regress = c("percent.mt"))

SRR783_val

###perform linear dimensional reduction###

SRR783_val <- RunPCA(SRR783_val, features = VariableFeatures(object = SRR783_val))

print(SRR783_val[["pca"]], dims = 1:5, nfeatures = 5)

#dev.off()

VizDimLoadings(SRR783_val, dims = 1:2, reduction = "pca")

ElbowPlot(SRR783_val,ndims = 50)

####cluster the cells###

SRR783_val <- FindNeighbors(SRR783_val, dims = 1:30)

####Run non-linear dimensional reduction (UMAP/tSNE)###

SRR783_val <- RunUMAP(SRR783_val, dims = 1:30)

SRR783_val<-RunTSNE(SRR783_val,dims=1:30)

SRR783_val <- FindClusters(SRR783_val, resolution = 0.6)

}

SRR783_val@meta.data[1:5,]

####因为前面跑过，所以直接从这步开始计算###

SRR783_val_db<-SRR783_val

sweep.res.list_SRR783_val <- paramSweep_v3(SRR783_val_db, PCs = 1:30)

sweep.stats_SRR783_val <- summarizeSweep(sweep.res.list_SRR783_val, GT = FALSE)

bcmvn_SRR783_val<-find.pK(sweep.stats_SRR783_val)

pK_value <- as.numeric(as.character(bcmvn_SRR783_val$pK[bcmvn_SRR783_val$BCmetric == max(bcmvn_SRR783_val$BCmetric)]))

annotations <- SRR783_val_db@meta.data$RNA_snn_res.0.6

homotypic.prop <- modelHomotypic(annotations) ####get the estimated number

nExp_poi <- round(homotypic.prop*length(SRR783_val_db@meta.data$orig.ident))

nExp_poi.adj <- round(nExp_poi*(1-homotypic.prop))

pN_value <- 0.25

pANN_value <- paste0("pANN_",pN_value,"_",pK_value,'_',nExp_poi)

SRR783_val_db <- doubletFinder_v3(SRR783_val_db, PCs = 1:30, pN = pN_value, pK = pK_value, nExp = nExp_poi, reuse.pANN = FALSE)

SRR783_val_db <- doubletFinder(SRR783_val_db, PCs = 1:30, pN = pN_value, pK = pK_value, nExp = nExp_poi.adj, reuse.pANN = pANN_value)

SRR783_val_db@meta.data[1:5,]

SRR783_val$doublets<-SRR783_val_db$DF.classifications_0.25_0.08_330

SRR783_val@meta.data[1:5,]

####saveRDS###

saveRDS(SRR783_val,file = "./SRR83_outs/SRR783_val.rds")

DimPlot(SRR783_val,reduction = "tsne",group.by = "doublets")

####merge the scRNA-data####

rm(list=ls())

library(Seurat)

library(dplyr)

library(future)

library(future.apply)

library(dplyr)

library(msigdbr)

library(clusterProfiler)

plan("multiprocess", workers = 8) ###set the compute core

options(future.globals.maxSize = 12000 * 1024^2)

getwd()

setwd("~/Documents_PC/scRNA-seq/Data/10X")

SRR780<-readRDS("./CC4/CC4_val.rds")

SRR781<-readRDS("./CC1/CC1_val.rds")

SRR782<-readRDS("./CC2/CC2_val.rds")

SRR783<-readRDS("./CC3/CC3_val.rds")

SRR783@meta.data[1:5,]

#####取小的对象###如果电脑内存较小的话只用1000个细胞，这里我们不用

SRR780<-SRR780[,1:1000]

SRR781<-SRR781[,1:1000]

SRR782<-SRR782[,1:1000]

SRR783<-SRR783[,1:1000]

##########

sce.mergeTEN<- merge(SRR780,y=c(SRR781,SRR782,SRR783),project = "scTEN")

sce.mergeTEN@meta.data[1:5,]

rm(SRR780,SRR781,SRR782,SRR783)

gc()

#saveRDS(sce.mergeTEN,file="./sce.mergeTEN_2.rds")

sce.mergeTEN<-readRDS(file="./sce.mergeTEN.rds")

####integration with harmony####

library(devtools)

#install_github("immunogenomics/harmony")

library(harmony)

gc()

sce.mergeTEN@meta.data[1:5,]

s.genes <- cc.genes$s.genes

g2m.genes <- cc.genes$g2m.genes

sce.mergeTEN <- CellCycleScoring(sce.mergeTEN, s.features = s.genes, g2m.features = g2m.genes, set.ident = TRUE)

sce.mergeTEN@meta.data[1:5,]

sce.mergeTEN<-NormalizeData(sce.mergeTEN,verbose = T)

sce.mergeTEN<-FindVariableFeatures(sce.mergeTEN,selection.method = "vst", nfeatures = 2000)

sce.mergeTEN<-ScaleData(sce.mergeTEN,vars.to.regress = c("percent.mt","S.Score","G2M.Score"),verbose = FALSE)

sce.mergeTEN<-RunPCA(sce.mergeTEN,verbose = T,npcs = 50)

ElbowPlot(sce.mergeTEN,ndims = 10)

p1 <- DimPlot(object = sce.mergeTEN, reduction = "pca", pt.size = .1, group.by = "orig.ident")

p2 <- VlnPlot(object = sce.mergeTEN, features = "PC_1", group.by = "orig.ident", pt.size = .1)

CombinePlots(plots=list(p1,p2))

sce.mergeTEN<-RunHarmony(sce.mergeTEN,group.by.vars = c("orig.ident"), plot_convergence = TRUE)

harmony_embeddings <- Embeddings(sce.mergeTEN, 'harmony')

dim(harmony_embeddings)

p3 <- DimPlot(object = sce.mergeTEN, reduction = "harmony", pt.size = .1, group.by = "orig.ident")

p4 <- VlnPlot(object = sce.mergeTEN, features = "harmony_1", group.by = "orig.ident", pt.size = .1)

CombinePlots(plots=list(p3,p4))

p1+p3

sce.mergeTEN <- sce.mergeTEN %>%

RunUMAP(reduction = "harmony", dims = 1:50) %>%

RunTSNE(reduction = "harmony", dims = 1:50) %>%

FindNeighbors(reduction = "harmony", dims = 1:50)

sce.mergeTEN<-FindClusters(sce.mergeTEN,resolution = 0.1)

#saveRDS(sce.mergeTEN,file="./sce.mergeTEN_2.rds")

#sce.mergeTEN<-readRDS(file="./sce.mergeTEN_2.rds")

table(Idents(sce.mergeTEN))

#Idents(sce_inter)<-sce_inter$seurat_clusters

sce.mergeTEN@meta.data[1:5,]

p1<-DimPlot(sce.mergeTEN,reduction = "tsne",label = T)

p1

DimPlot(sce.mergeTEN,reduction = "umap",label = T)

DimPlot(sce.mergeTEN,reduction = "tsne",label = T,split.by = "tissue_type")

DimPlot(sce.mergeTEN,reduction = "umap",label = T,split.by = "tissue_type")

table(sce.mergeTEN@meta.data$cell_cluster_origin)

sce.mergeTEN@meta.data[1:5,]

p2<-DimPlot(sce.mergeTEN,reduction = "tsne",group.by = "cluster",label = T)

p2

DimPlot(sce.mergeTEN,reduction = "tsne",group.by = "cluster",label = T)

DimPlot(sce.mergeTEN,group.by = "cell_predict",reduction = "tsne",label = T)

table(Idents(sce.mergeTEN),sce.mergeTEN$cell_cluster_origin)

DimPlot(sce.mergeTEN,reduction = "tsne",label = T)

####FPI

#### LPCAT3、ACSL4、NCOA4、ALOX15、GPX4

FeaturePlot(sce.mergeTEN,features = c("LPCAT3","ACSL4","NCOA4","ALOX15","GPX4"),reduction = "tsne",label = T)

###epithelial cell

FeaturePlot(sce.mergeTEN,features = c("EPCAM","CDH1","KRT7","KRT5","UPK1A","KRT20"),reduction = "tsne",label = T)

##TNK cell

FeaturePlot(sce.mergeTEN,features = c("CD3D","CD8A","CD4","NCAM1","IL7R"),reduction = "tsne",label = T)

##endothelial cell

FeaturePlot(sce.mergeTEN,features = c("VWF","PECAM1"),reduction = "tsne",label = T)

####fibroblasts and pericytes

FeaturePlot(sce.mergeTEN,features = c("RGS5","COL1A1","PDGFRA","PDGFRB","DES","FAP"),reduction = "tsne",label = T)

###B cell and plasma

FeaturePlot(sce.mergeTEN,features = c("CD19","JCHAIN","CD79A"),reduction = "tsne",label = T)

###myeloid cell

FeaturePlot(sce.mergeTEN,features = c("CD74","CD14","FCGR3A","S100A8"),reduction = "tsne",label = T)

###mast cell

FeaturePlot(sce.mergeTEN,features = c("CPA3"),reduction = "tsne",label = T)

####差异基因

marker <- FindMarkers(sce.mergeTEN, ident.1 = "Tumor",ident.2 = "Normal",group.by="tissue_type")

sig_dge.cluster <- subset(marker, p_val_adj<0.05&abs(avg_log2FC)>1)

####marker gene

sce.mergeTEN.markers <- FindAllMarkers(sce.mergeTEN, only.pos = TRUE, min.pct = 0.25, logfc.threshold = 0.25)

write.csv(sce.mergeTEN.markers,"./sce.mergeTEN.markers.csv")

marker <- FindMarkers(sce.mergeTEN, ident.1 = "21",ident.2 = "8",only.pos = TRUE, min.pct = 0.25, logfc.threshold = 0.25)

c<-table(Idents(sce.mergeTEN),sce.mergeTEN$cell_predict)

c

write.csv(c,file="../10X/cc.csv")

p1+p2

sce.mergeTEN<-RenameIdents(sce.mergeTEN,"0"="T cell","2"="Epithelial","1"="Smooth muscle cell","6"="Epithelial","3"="Fibroblasts","4"="Endothelial cell","7"="Macrophage","8"="Epithelial",

"9"="B cell","10"="Lymphocyte","11"="Lymphocyte","5"="Plasma cell")

sce.mergeTEN<-RenameIdents(sce.mergeTEN,"Fibroblasts"="Fibroblasts")

DimPlot(sce.mergeTEN,reduction = "tsne",label = T)

DimPlot(sce.mergeTEN,reduction = "umap",label = T)

#table(Idents(sce.mergeTEN))

DimPlot(sce.mergeTEN,reduction = "umap",label = T,group.by = "celltype")

sce.mergeTEN$Seurat_harmony<-Idents(sce.mergeTEN)

sce.mergeTEN@meta.data[1:5,]

markers<-FindAllMarkers(sce.mergeTEN,only.pos = T,min.pct = 0.25,logfc.threshold = 0.25)

write.csv(markers,file="./markers.csv")

library(ggsci)

##define the color

??ggsci

#pal_npg()(11)

cors<-pal_npg()(7)

cors<-c("#029149","#E0367A","#5D90BA","#431A3D","#FFD121","#D8D155","#223D6C","#D20A13","#f27024")

##Reduction plot##

DimPlot(sce.mergeTEN,reduction="tsne",cols=cors,label = T,pt.size = 0.8)

DimPlot(sce.mergeTEN,reduction="umap",cols=cors)

###By sample

DimPlot(sce.mergeTEN,reduction="tsne",cols = cors,split.by = "orig.ident",ncol = 4)

DimPlot(sce.mergeTEN,reduction="umap",cols = cors,split.by = "orig.ident",ncol = 4)

###By type

sce.mergeTEN$tissue_type

DimPlot(sce.mergeTEN,reduction="umap",cols = cors,split.by = "tissue_type")

DimPlot(sce.mergeTEN,reduction="tsne",cols = cors,group.by = "tissue_type",pt.size = 0.8)

#DimPlot(sce_inter,reduction="umap",cols = cors,label = F)

#DimPlot(sce_inter,reduction="umap",cols = cors,split.by = "orig.ident",ncol = 3)

#markers <- FindAllMarkers(sce.mergeTEN, only.pos = TRUE, min.pct = 0.25, logfc.threshold = 0.25)

write.csv(markers,file="./markers.csv")

####vlnplot and dotplot#####

features = c("CD3G","CD8A","KRT19","KRT7","KRT18","ACTA2","COL1A1","PDGFRA","FAP","VWF","PECAM1","CD14","CD68","CD37","CD74","KIT","JCHAIN")

length(features)

VlnPlot(sce.mergeTEN,features = features[1:6],cols = cors,pt.size = 0)

VlnPlot(sce.mergeTEN,features = features[7:12],cols = cors,pt.size = 0)

VlnPlot(sce.mergeTEN,features = features[12:17],cols = cors,pt.size = 0)

#VlnPlot(sce.mergeTEN,features = features[18:20],cols = cors,pt.size = 0)

VlnPlot(sce.mergeTEN,features = c("CD14","VEGFA"),cols = cors,pt.size = 0)

DotPlot(sce.mergeTEN,features = features,cols = c("grey","darkblue"))

#####distribution plot

library(ggplot2)

table(sce.mergeTEN$tissue_type,sce.mergeTEN$orig.ident)

tab<-table(Idents(sce.mergeTEN),sce.mergeTEN$orig.ident)

tab

tab<-as.data.frame(tab)

tab

ggplot(tab,aes(x=Var2,y=Freq,fill=Var1))+

geom_bar(stat='identity',position='stack',alpha=.5)+

labs(title='',x='group',y='Cell Number')+

theme(legend.justification = 'right',

legend.position = 'right',

legend.key.height = unit(0.1,'cm'),

panel.background = element_blank(),

axis.line=element_line(size=0.5,colour="black")

)+ scale_fill_manual(values=cors)

tab<-table(Idents(sce.mergeTEN),sce.mergeTEN$orig.ident)

tab

tab<-prop.table(tab,2)*100

tab

tab<-as.data.frame(tab)

ggplot(tab,aes(x=Var2,y=Freq,fill=Var1))+

geom_bar(stat='identity',position='stack',alpha=.5)+

labs(title='',x='group',y='Cell Proportion (%)')+

theme(legend.justification = 'right',

legend.position = 'right',

legend.key.height = unit(0.1,'cm'),

panel.background = element_blank(),

axis.line=element_line(size=0.5,colour="black")

)+ scale_fill_manual(values=cors)

####compare the distribution of CD45+

tab<-table(sce.mergeTEN$orig.ident,Idents(sce.mergeTEN))

tab

tab<-tab[,2:6]

tab

tab<-prop.table(tab,1)*100

tab

tab<-as.data.frame(tab)

tab

group<-c(rep(c("Normal","Tumor"),5,each=2))

group

tab$group=group

tab

tab[tab$Var2=="T/NK",]

library(ggpubr)

p <- ggboxplot(tab[tab$Var2=="T/NK",], x = "group", y = "Freq",

color = "group", palette =c("#00AFBB", "#E7B800"),###定义颜色

add = "jitter", shape = "group")

p

my_comparisons <- list( c("Tumor","Normal") )

p + stat_compare_means(comparisons = my_comparisons,method = "t.test",paired = F)+ # Add pairwise comparisons p-value

stat_compare_means(label.y = 70,method = "t.test",paired = F)

tab

tab[tab$Var2=="B",]

p <- ggboxplot(tab[tab$Var2=="B",], x = "group", y = "Freq",

color = "group", palette ="npg",###定义颜色

add = "jitter", shape = "group")

p

my_comparisons <- list( c("Tumor","Normal") )

p + stat_compare_means(comparisons = my_comparisons,method = "t.test",paired = F)+ # Add pairwise comparisons p-value

stat_compare_means(label.y = 25,method = "t.test",paired = F)

sce.mergeTEN@meta.data[1:5,]

saveRDS(sce.mergeTEN,file = "./sce.mergeTEN.rds")

save.image(file="./SCE_TEN.RData")###保存镜像

load(file="./SCE_TEN.RData")

####第一天课程结束点，请大家联系其余几个datasets的分析流程####

#### 第二天课程开始####

markers<-read.csv(file="./sce.mergeTEN.markers.csv",row.names=1)

#Doheatmap#####

features<-unique(markers$gene[markers$p_val_adj<0.0001&markers$avg_log2FC>1.5]) ####通常p_val_adj<0.05,avg_log2FC>0.25

features

####end####

getwd()

#sce.mergeTEN<-ScaleData(sce.mergeTEN, vars.to.regress = c("percent.mt","S.Score","G2M.Score"))

sce.mergeTEN_DR<- ScaleData(sce.mergeTEN, vars.to.regress = c("percent.mt","S.Score","G2M.Score"),features = features)

pdf(file="DO_heatmap.pdf",width = 8,height = 10)

DoHeatmap(sce.mergeTEN_DR,features = features,group.colors = cors)###It take several mins####

dev.off()

#cors

####matrix

genemeanMatrix<-AverageExpression(sce.mergeTEN)

genemeanMatrix<-genemeanMatrix$RNA

features_to_anno = c("CD3G","CD8A","KRT19","KRT7","KRT18","ACTA2","COL1A1","PDGFRA","FAP","VWF","PECAM1","CD14","CD68","CD37","CD74","KIT","JCHAIN")

length(features_to_anno)

geneMatrix<-genemeanMatrix[features_to_anno,]

###rownames

cc<-rownames(geneMatrix)

cc

#cc[which(!(cc%in%features_to_anno))]<-""

#cc

table(Idents(sce.mergeTEN))

levels(Idents(sce.mergeTEN))##知道细胞亚群包含哪些

####information for columns

annotation_col = data.frame(

CellType = c("T cell","Epithelial","Smooth muscle cell","Fibroblasts","Endothelial cell","Macrophage","B cell","Lymphocyte","Plasma cell")

)

rownames(annotation_col) = colnames(geneMatrix)

####define the seq

annotation_col$CellType<-factor(annotation_col$CellType,levels= c("T cell","Epithelial","Smooth muscle cell","Fibroblasts","Endothelial cell","Macrophage","B cell","Lymphocyte","Plasma cell"))

levels(Idents(sce.mergeTEN))

cors

paste(c("T cell","Epithelial","Smooth muscle cell","Fibroblasts","Endothelial cell","Macrophage","B cell","Lymphocyte","Plasma cell"),cors,sep='"="')

####color to use in heatmap

ann_colors = list(

CellType = c("T cell"="#029149" , "Epithelial"="#E0367A" ,

"Smooth muscle cell"="#5D90BA" ,"Fibroblasts"="#431A3D",

"Endothelial cell"="#FFD121" , "Macrophage"="#D8D155" ,

"B cell"="#223D6C" , "Lymphocyte"="#D20A13",

"Plasma cell"="#f27024" )

#GeneClass = c("CD4+" = "#7570B3", "CD8+" = "#E7298A","NK" = "#66A61E")

)

?pheatmap

library(RColorBrewer)

#dev.off()

#dev.new()

ComplexHeatmap::pheatmap(as.matrix(geneMatrix), annotation_col = annotation_col,cluster_rows = F,labels_row = cc,

annotation_colors = ann_colors,cluster_cols = F,scale = "row",color = colorRampPalette(rev(brewer.pal(n = 11, name ="RdYlBu")))(50))

#gaps_row = c(8, 14) )

#####GSva analysis######

#####GSEA analysis####

library(devtools)

install_github("arc85/singleseqgset")##https://arc85.github.io/singleseqgset/articles/singleseqgset.html

#library(singleseqgset)

library(singleseqgset)

?msigdbr

h.human <- msigdbr(species="Homo sapiens",category="H")

h.names <- unique(h.human$gs_name)

h.sets <- vector("list",length=length(h.names))

h.names

names(h.sets) <- h.names

for (i in names(h.sets)) {

h.sets[[i]] <- pull(h.human[h.human$gs_name==i,"gene_symbol"])

}

h.sets

#expr.mat=sce.mergeTEN@assays$RNA@data

expr.mat[1:5,1:5]

sce.mergeTEN@meta.data[1:5,]

###内存小的话，跑小样本

expr.mat=sce.mergeTEN@assays$RNA@data#[,c(1:1000)]###如果用全部细胞胞，则不要取前面1000个细胞

expr.mat[1:5,1:5]

group=sce.mergeTEN$Seurat_harmony#[1:1000]##如果用全部细胞胞，则不要取前面1000个细胞

group

####计算每组相对于其他组的显著差异基因

logfc.data <- logFC(cluster.ids=sce.mergeTEN$Seurat_harmony,expr.mat=expr.mat)

#logfc.data <- logFC(cluster.ids=sce.mergeTEN$Seurat_harmony[1:1000],expr.mat=expr.mat[,c(1:1000)])

names(logfc.data)

gse.res <- wmw_gsea(expr.mat=expr.mat,cluster.cells=logfc.data[[1]],log.fc.cluster=logfc.data[[2]],gene.sets=h.sets)

names(gse.res)

res.stats <- gse.res[["GSEA_statistics"]]

res.stats

ac<-data.frame(cluster=factor(colnames(res.stats)))

ac

rownames(ac)=colnames(res.stats)

ann_colors = list(

cluster = c("T cell"="#029149" , "Epithelial"="#E0367A" ,

"Smooth muscle cell"="#5D90BA" ,"Fibroblasts"="#431A3D",

"Endothelial cell"="#FFD121" , "Macrophage"="#D8D155" ,

"B cell"="#223D6C" , "Lymphocyte"="#D20A13",

"Plasma cell"="#f27024" )

#GeneClass = c("CD4+" = "#7570B3", "CD8+" = "#E7298A","NK" = "#66A61E")

)

#cors

#dev.off()

library(stringr)

rownames(res.stats)<-str_replace_all(rownames(res.stats),pattern = "HALLMARK_",replacement ="")

pheatmap::pheatmap(res.stats,fontsize_row = 8,annotation_col = ac,annotation_legend = F,annotation_colors = ann_colors,cluster_rows = T,

cluster_cols = F,scale = "row")

#ggsave("GSEAheatmap.pdf",width = 22,height = 18,units = "cm")

#####compare the differentially expressed genes between the sunclusters#####

table(Idents(sce.mergeTEN))

Idents(sce.mergeTEN)

Bcells<-Idents(sce.mergeTEN)%in%c("B cell","Plasma cell")

table(Bcells)

Bcells<-sce.mergeTEN[,Bcells]

table(Idents(Bcells))

#####compare the gene expression between the cells####

bcmarkers<-FindAllMarkers(Bcells,only.pos = T,min.pct = 0.1,logfc.threshold = 0)

library(dplyr)

library(msigdbr)

library(clusterProfiler)

#m_t2g <- msigdbr(species = "Homo sapiens", category = "C5",subcategory = "BP")%>%dplyr::select(gs_name, human_gene_symbol)

marker<-bcmarkers[bcmarkers$p_val_adj<0.05,]

library(stringr)

#BiocManager::install("org.Hs.eg.db")

#marker$gene<-str_replace_all(marker$gene,pattern = "[.]",replacement = "-")

cellID<-bitr(marker$gene,fromType = "SYMBOL",toType = "ENTREZID",OrgDb = "org.Hs.eg.db")

cellID

tmp<-marker$gene%in%cellID$SYMBOL

table(tmp)

markers_2<-marker[tmp,]

table(markers_2$gene%in%cellID$SYMBOL)

?left_join

library(dplyr)

mmm<-left_join(markers_2,cellID,by=c("gene"="SYMBOL"))

?compareCluster

xx.formula <- compareCluster(ENTREZID~cluster, data=mmm,

fun="enrichKEGG")

xx.formula_GO <- compareCluster(ENTREZID~cluster, data=mmm,OrgDb='org.Hs.eg.db',

fun="enrichGO")

dotplot(xx.formula,showCategory =20)

dotplot(xx.formula_GO,showCategory = 10)

dd<-as.data.frame(xx.formula)

dd<-dd[c(2,5,6,15,17,21,22,30,42:48),]

dd$logp<- -1*log10(dd$pvalue)

#cors[4:6]

library(ggpubr)

#head(dd)

#dd$Description<-paste0(1:15,dd$Description)

dd$cluster<-factor(dd$cluster,levels=c("B","Plasma_B"))

ggbarplot(dd, x = "Description", y = "logp",

fill = "cluster", # change fill color by mpg_level

color = "white", # Set bar border colors to white

palette = "jco",#c("#D62728FF", "#8C564BFF"), # jco journal color palett. see ?ggpar

sort.val = "asc", # Sort the value in descending order

sort.by.groups = T, # Don't sort inside each group

x.text.angle = 0, # Rotate vertically x axis texts

ylab = "-log10(pvalue)",

legend.title = "KEGG terms",

rotate = TRUE,

ggtheme = theme_classic()

)

save.image(file="./SCE_TEN.RData")

load(file="./SCE_TEN.RData")

####merge the scRNA-data####

rm(list=ls())

library(Seurat)

library(dplyr)

library(future)

library(future.apply)

library(monocle)

library(ggsci)

plan("multicore", workers = 4) ###set the compute core

options(future.globals.maxSize = 15000 * 1024^2)

#getwd()

Myeloid<-readRDS("./CAFsMyeloid.rds")

table(Idents(sce.mergeTEN))

Myeloid<-sce.mergeTEN[,Idents(sce.mergeTEN)%in%c("Fibroblasts")]#Idents(sce.mergeTEN)=="B"｜Idents(sce.mergeTEN)=="Plasma_B"

rm(sce.mergeTEN)

gc()

###check the

s.genes <- cc.genes$s.genes

g2m.genes <- cc.genes$g2m.genes

Myeloid <- CellCycleScoring(Myeloid, s.features = s.genes, g2m.features = g2m.genes, set.ident = TRUE)

Myeloid@meta.data[1:5,]

Myeloid<-NormalizeData(Myeloid,verbose = T)

Myeloid<-FindVariableFeatures(Myeloid,selection.method = "vst", nfeatures = 2000)

Myeloid<-ScaleData(Myeloid,vars.to.regress = c("percent.mt","S.Score","G2M.Score"),verbose = FALSE)

Myeloid<-RunPCA(Myeloid,verbose = T,npcs = 50)

ElbowPlot(Myeloid,ndims = 50)

p1 <- DimPlot(object = Myeloid, reduction = "pca", pt.size = .1, group.by = "orig.ident")

p2 <- VlnPlot(object = Myeloid, features = "PC_1", group.by = "orig.ident", pt.size = .1)

CombinePlots(plots=list(p1,p2))

library(harmony)

Myeloid<-RunHarmony(Myeloid,"orig.ident", plot_convergence = TRUE)

harmony_embeddings <- Embeddings(Myeloid, 'harmony')

dim(harmony_embeddings)

p3 <- DimPlot(object = Myeloid, reduction = "harmony", pt.size = .1, group.by = "orig.ident")

p4 <- VlnPlot(object = Myeloid, features = "harmony_1", group.by = "orig.ident", pt.size = .1)

CombinePlots(plots=list(p3,p4))

p1+p3

Myeloid <- Myeloid %>%

RunUMAP(reduction = "harmony", dims = 1:50) %>%

RunTSNE(reduction = "harmony", dims = 1:50) %>%

FindNeighbors(reduction = "harmony", dims = 1:50)

Myeloid<-FindClusters(Myeloid,resolution = 0.1)

DimPlot(Myeloid,reduction = "tsne",label = T)

DimPlot(Myeloid,reduction = "umap",label = T)

cors<-pal_npg()(10)

DimPlot(Myeloid,reduction = "tsne",label = T,split.by = "tissue_type")

###Monocytes

#iCAFs

cors<-c("grey","red")

FeaturePlot(Myeloid,features = c("C7","CNN1"),reduction = "tsne",label = F,cols=cors)

###vCAFs

FeaturePlot(Myeloid,features = c("TNXB","CTGF"),reduction = "tsne",label = F,cols=cors)

##mCAFs

FeaturePlot(Myeloid,features = c("SELENOM","RACK1"),reduction = "tsne",label = F,cols=cors)

Myeloid<-RenameIdents(Myeloid,"1"="mCAFs","3"="mCAFs","5"="iCAFs","7"="mCAFs","8"="vCAFs","4"="iCAFs",

"9"="mCAFs","0"="vCAFs","6"="iCAFs","2"="mCAFs","10"="mCAFs")

library(ggsci)

cors<-pal_npg()(2)

cors<-c("#029149","#E0367A","#5D90BA","#431A3D","#FFD121","#D8D155","#223D6C","#D20A13","#f27024",cors)

##Reduction plot##

DimPlot(Myeloid,reduction="tsne",cols=cors)

DimPlot(Myeloid,reduction="umap",group.by="RNA_snn_res.0.1",cols=cors)

#Myeloid<-readRDS("./Myeloid.rds")

mmt<-Myeloid[,Idents(Myeloid)%in%c("mCAFs","iCAFs","vCAFs")]

###monocle analysis of the trajectory###

library(monocle)

##get the monoand macro###BDonly

###更多的信息查看http://cole-trapnell-lab.github.io/monocle-release/docs/#installing-monocle

mmt$cell_type_val<-Idents(mmt)

mmt@meta.data[1:5,]

mmt<-FindVariableFeatures(mmt)

?FindVariableFeatures

matrix<-as.matrix(mmt@assays$RNA@counts)

dim(matrix)

matrix[1:500,1:10]

gene_ann <- data.frame(

gene_short_name = row.names(matrix),

row.names = row.names(matrix)

)##基因注释

#oct[["cell_group"]]<-Idents(oct)

mmt@meta.data[1:5,]

sample_ann <- mmt@meta.data###细胞注释

fd <- new("AnnotatedDataFrame",data=gene_ann)

pd<-new("AnnotatedDataFrame",data=sample_ann)

#?newCellDataSet

sc_cds_2 <- newCellDataSet(matrix, phenoData = pd,featureData =fd,expressionFamily = negbinomial.size(),lowerDetectionLimit=0.01)

sc_cds_2 <- estimateSizeFactors(sc_cds_2)

sc_cds_2 <- estimateDispersions(sc_cds_2)

####QC#####

?detectGenes

sc_cds_2 <- detectGenes(sc_cds_2, min_expr = 1)

expressed_genes <- row.names(subset(fData(sc_cds_2), num_cells_expressed >= 5))

fData(sc_cds_2)[1:5,]

#####cluster analysis######

###以高变基因为ordering

#ordering_genes<-mmt@assays$RNA@var.features

###以两组之间差异基因为ordering gene

diff_test_res <- differentialGeneTest(sc_cds_2[expressed_genes,],

fullModelFormulaStr = "~cell_type_val",cores = 4,

verbose = T) #+num_genes_expressed+orig.ident

ordering_genes <- row.names(subset(diff_test_res, qval < 1e-2)) #1e-200

#ordering_genes<-markers %>% group_by(cluster) %>% top_n(n=200,wt=avg_logFC)

sc_cds2 <- setOrderingFilter(sc_cds_2, ordering_genes)

plot_ordering_genes(sc_cds2)

sc_cds2<- reduceDimension(sc_cds2, max_components = 2, num_dim=6,reduction_method = "DDRTree")#,residualModelFormulaStr = "~orig.ident")

sc_cds2 <- orderCells(sc_cds2)

plot_cell_trajectory(sc_cds2, color_by = "cell_type_val",show_branch_points = T)

#sc_cds2 <- orderCells(sc_cds2,root_state = 8)

#pData(sc_cds_2)[1:5,]

#p1<-plot_cell_trajectory(sc_cds2, color_by = "RNA_snn_res.0.8",show_branch_points = F)#+facet_wrap(~RNA_snn_res.0.8)

plot_cell_trajectory(sc_cds2, color_by = "State",show_branch_points = T)#+facet_wrap(~Site)

plot_cell_trajectory(sc_cds2, color_by = "Pseudotime",show_branch_points = T)

plot_cell_trajectory(sc_cds2, markers = c("COL6A3","C3","IL6"),use_color_gradient = T,show_branch_points = F)

to_be_tested <- row.names(subset(fData(sc_cds2), gene_short_name %in% c("COL6A3","C3","IL6")))

cds_subset <- sc_cds2[to_be_tested,]

plot_genes_in_pseudotime(cds_subset, color_by = "cell_type_val")

####get the genes change along with the Pseudotime###

diff_test_res <- differentialGeneTest(sc_cds2[expressed_genes,],

fullModelFormulaStr = "~sm.ns(Pseudotime)",cores = 5)

sig_gene_names <- row.names(subset(diff_test_res, qval < 0.05))

#dev.off()

dev.new()

pseudoplot<-plot_pseudotime_heatmap(sc_cds2[sig_gene_names,],

num_clusters = 5,###进行调节分群

cores = 5,

show_rownames = F,return_heatmap = T)

####get the gene names in each cluster##

clusters <- cutree(pseudoplot$tree_row, k = 4)

clustering <- data.frame(clusters)

clustering[,1] <- as.character(clustering[,1])

colnames(clustering) <- "Gene_Clusters"

table(clustering)

library(msigdbr)

library(clusterProfiler)

msigdbr_show_species()

m_df <- msigdbr(species = "Homo sapiens")

m_t2g_C5 <- msigdbr(species = "Homo sapiens", category = "C5",subcategory = "BP") %>%

dplyr::select(gs_name, gene_symbol)

m_t2g_C7 <- msigdbr(species = "Homo sapiens", category = "C7") %>%

dplyr::select(gs_name, gene_symbol)

m_t2g_C2 <- msigdbr(species = "Homo sapiens", category = "C2",subcategory = c("CP:KEGG")) %>%

dplyr::select(gs_name, gene_symbol) ###signating pathways

###group1##

gene1<-rownames(clustering)[clustering[,1]==1]

em_group1 <- enricher(gene1, TERM2GENE=m_t2g_C5)

em_group_pathway_1 <- enricher(gene1, TERM2GENE=m_t2g_C2)

###simplify(em_group1)

head(em_group1)

head(em_group_pathway_1)

barplot(em_group1,showCategory = 20)

barplot(em_group_pathway_1)

write.csv(em_group1,file="./heatmap_cluster1_enrichment.csv")

gene2<-rownames(clustering)[clustering[,1]==2]

em_group2<- enricher(gene2, TERM2GENE=m_t2g_C5)

head(em_group2)

barplot(em_group2,showCategory = 20)

write.csv(em_group2,file="./heatmap_cluster2_enrichment.csv")

gene3<-rownames(clustering)[clustering[,1]==3]

em_group3<- enricher(gene3, TERM2GENE=m_t2g_C5)

head(em_group3)

barplot(em_group3,showCategory = 20)

write.csv(em_group3,file="./heatmap_cluster3_enrichment.csv")

gene4<-rownames(clustering)[clustering[,1]==4]

em_group4<- enricher(gene4, TERM2GENE=m_t2g_C5)

head(em_group4)

barplot(em_group4,showCategory = 20)

write.csv(em_group4,file="./heatmap_cluster4_enrichment.csv")

saveRDS(Myeloid,file="./CAFsMyeloid.rds")

rm(list=ls())

library(Seurat)

library(dplyr)

library(future)

library(future.apply)

library(stringr)

library(infercnv)

plan("multiprocess", workers = 6) ###set the compute core

options(future.globals.maxSize = 60000 * 1024^2)

getwd()

rm(list=ls())

sce.mergeTEN<-readRDS("./sce.mergeTEN_2.rds")

table(Idents(sce.mergeTEN))

epith<-sce.mergeTEN[,Idents(sce.mergeTEN)=="Epithelial"]

rm(sce.mergeTEN)

#####选择原来1/5的细胞数进行分析###

se<-seq(from=1,to=3491,by=1)

epith<-epith[,se]###缩小了的epithel数据，正式分析时不要取这个，直接用epith

####

matrix<-as.matrix(epith@assays$RNA@counts)

table(scenicOptions$features)

epith@meta.data[1:5,]

ann<-as.data.frame(ifelse(epith@meta.data[,"tissue_type"]=="Tumor","maglignant_Epithelial","Normal"),row.names=rownames(epith@meta.data))

ann<-as.matrix(ann)

colnames(ann)<-"cell_type"

gene.order<-read.table(file="./gene_position_updated_hg19.txt",header = F,sep = "\t")

rownames(gene.order)<-gene.order$V1

gene.order<-gene.order[,-1]

inferobj<-CreateInfercnvObject(raw_counts_matrix=matrix,

annotations_file=ann,

gene_order_file=gene.order,

ref_group_names=c("Normal"))

outdir_1<-"./epi_3"####输出的文件夹位置

#####会花比较长的时间，课上不跑，留在下课后跑

infercnv_obj = infercnv::run(inferobj,

cutoff=0.1, # cutoff=1 works well for Smart-seq2, and cutoff=0.1 works well for 10x Genomics

out_dir=outdir_1,

cluster_by_groups=TRUE,

denoise=TRUE,plot_steps = T,

HMM=F)

####完整命令行

inferobj <- infercnv::run(inferobj,

cutoff=0.1,

out_dir=outdir_1,

window_length = 101,

max_centered_threshold = 3,

plot_steps = F,denoise = TRUE,

cluster_by_groups=T,

no_prelim_plot = T,

HMM=FALSE,

sd_amplifier = 1.3,

analysis_mode = "samples",

num_threads=3,

png_res = 200)

####check the malignant cells#####

####load the package###

library(cluster)

library(scales)

library(Seurat)

library(reshape2)

library(ggplot2)

library(cowplot)

library(fpc)

library(dplyr)

library(cluster)

#install.packages("fpc")

outdir<-"./epi_3"##改为保存infercnv.22_denoised.observations.txt文件的位置

infercnv_output <- as.data.frame(t(read.table(paste0(outdir,

"/infercnv.observations.txt"))))

library(stringr)

rownames(infercnv_output)<-str_replace_all(rownames(infercnv_output),pattern = "[.]",replacement = "-")##因为inferCNV将-转变为.了

infercnv_output_ref <- as.data.frame(t(read.table(paste0(outdir,

"/infercnv.references.txt"))))

rownames(infercnv_output_ref)<-str_replace_all(rownames(infercnv_output_ref),pattern = "[.]",replacement = "-")

saveRDS(ann,file="ann.rds")

ann<-readRDS(file="ann.rds")

ann[1:5,]

metadata_df<-data.frame(ann)

dim(metadata_df)

metadata_df$cell_ids<-rownames(metadata_df)

epithelial_ids <- metadata_df$cell_ids[grep("magli", metadata_df$cell_type)]

epithelial_heatmap <- infercnv_output[rownames(infercnv_output) %in% epithelial_ids,]

tme_ids <- metadata_df$cell_ids[grep("magli", metadata_df$cell_type,invert = T)]

tme_heatmap<-infercnv_output_ref[rownames(infercnv_output_ref)%in% tme_ids,]

print(paste0("Number of heatmap rows after non-epithelial thrown: ",

nrow(epithelial_heatmap)))

#

epithelial_metadata <- metadata_df[rownames(epithelial_heatmap),]

tme_metadata <- metadata_df[rownames(tme_heatmap),]

library(scales)

scaled_df <- as.data.frame(rescale(as.matrix(epithelial_heatmap), c(-1,1)))

CNA_values <- apply(scaled_df, 1, function(y) {

return(mean(y^2))

})

tme_scaled_df <- as.data.frame(rescale(as.matrix(tme_heatmap), c(-1,1)))

tme_CNA_values <- apply(tme_scaled_df, 1, function(y) {

return(mean(y^2))

})

CNA_value_df <- data.frame(

row.names = names(CNA_values),

CNA_value = CNA_values

)

tme_CNA_value_df <- data.frame(

row.names = names(tme_CNA_values),

CNA_value = tme_CNA_values

)

epithelial_metadata <- cbind(epithelial_metadata, CNA_value_df)

print(paste0(

"Are epithelial_metadata rownames still in the same order as epithelial_heatmap?? ",

identical(rownames(epithelial_heatmap), rownames(epithelial_metadata))

))

tme_metadata <- cbind(tme_metadata, tme_CNA_value_df)

total<-rbind(epithelial_heatmap,tme_heatmap)

total_df<-rbind(CNA_value_df,tme_CNA_value_df)

#table(rownames(total)==rownames(total_df))

###对肿瘤全部细胞CNV值进行排序 并取前面5%作为参照

CNA_order <- order(CNA_value_df$CNA_value, decreasing=T)

ordered_CNA_values <- data.frame(

row.names = rownames(CNA_value_df)[CNA_order],

CNA_value = CNA_value_df[CNA_order,]

)

top_cancer <- head(ordered_CNA_values, nrow(ordered_CNA_values)*0.05)###top5%的细胞

top_cancer

dim(top_cancer)

top_cancer_CNV_average <- apply(epithelial_heatmap[rownames(top_cancer),], 2, mean)

top_cancer_CNV_average

total_correlations <- apply(total, 1, function(x) {

cor <- cor.test(as.numeric(x), top_cancer_CNV_average, method = "pearson")

cor_result <- data.frame(cor$estimate, cor$p.value)

return(cor_result)

})

total_correlation_df <- do.call("rbind", total_correlations)

total_metadata<-rbind(epithelial_metadata,tme_metadata)

table(rownames(total_metadata)==rownames(total_correlation_df))

total_metadata <- cbind(total_metadata, total_correlation_df)

print(paste0(

"Are epithelial_metadata rownames still in the same order as epithelial_heatmap?? ",

identical(rownames(epithelial_heatmap), rownames(epithelial_metadata))

))

total_metadata<-data.frame(total_metadata)

total_metadata$normal_cell_call <- "Normal"

#####玉质需要自己设置

total_metadata$normal_cell_call[

total_metadata$CNA_value > 0.2|total_metadata$cor.estimate > 0.2

] <- "Cancer"

table(total_metadata$normal_cell_call,total_metadata$cell_type)###

#epithelial_metadata<-epithelial_metadata[,c(1:8)]

# create quad plot:

library(ggplot2)

p <- ggplot(total_metadata,

aes(x=CNA_value, y=cor.estimate, color=as.factor(normal_cell_call)))+

geom_point()+

scale_color_manual(values=c("black", "#74add1"),

labels=c("Cancer", "Normal"))+

xlab("Infercnv level")+

ylab("Corr. with top 5% cancer (p<0.05)")+

theme(legend.title = element_blank())+

geom_vline(xintercept = 0.2)+

geom_hline(yintercept = 0.2)

p

####定义为unassign####

total_metadata$normal_cell_call_2<-total_metadata$normal_cell_call

total_metadata$normal_cell_call_2[

total_metadata$cell_type=="maglignant_Epithelial"&total_metadata$normal_cell_call=="Normal"

] <- "Unassigned"

table(total_metadata$normal_cell_call_2)

p <- ggplot(total_metadata,

aes(x=CNA_value, y=cor.estimate, color=as.factor(normal_cell_call_2)))+

geom_point()+

scale_color_manual(values=c("black", "#74add1", "#b2182b"),

labels=c("Cancer", "Normal", "Unassigned"))+

xlab("Infercnv level")+

ylab("Corr. with top 5% cancer (p<0.05)")+

theme(legend.title = element_blank())+

geom_vline(xintercept = 0.04)+

geom_hline(yintercept = 0.2)

p

write.csv(total_metadata,file="./Epithelial_tumor_or_normal.csv")

save.image(file="./infCNV.RData")

rm(list=ls())

#install.packages("Seurat")

library(Seurat)

library(dplyr)

library(future)

library(future.apply)

plan("multiprocess", workers = 6) ###set the compute core

options(future.globals.maxSize = 60000 * 1024^2)

getwd()

library(stringr)

#memory.limit()

#BiocManager::install("igraph",force = T)

#setwd("~/Documents_PC/scRNA-seq/Data/")

####prepare the data for cce.mergeTellchat analysis####

sce.mergeTEN<-readRDS("./sce.mergeTEN_3.rds")

#write.table(as.matrix(sce.mergeTEN@assays$RNA@data), 'cellphonedb_count.txt', sep='\t', quote=F)

#meta_data <- cbind(rownames(sce.mergeTEN@meta.data), sce.mergeTEN@meta.data[,'Seurat_harmony', drop=F])

#meta_data <- as.matrix(meta_data)

#meta_dat[is.na(meta_data)] = "Unkown" # 细胞类型中不能有NA

#write.table(meta_data, 'cellphonedb_meta.txt', sep='\t', quote=F, row.names=F)

table(Idents(sce.mergeTEN))

sce.mergeTEN<-sce.mergeTEN[,Idents(sce.mergeTEN)!="Doublets"]

sce.mergeTEN@meta.data[1:5,]

table(sce.mergeTEN$tissue_type)

levels(Idents(sce.mergeTEN))

normaldata<-sce.mergeTEN[,sce.mergeTEN$tissue_type=="Normal"]

tumordata<-sce.mergeTEN[,sce.mergeTEN$tissue_type=="Tumor"]

##减少计算，只分析前面2000个细胞

normaldata<-normaldata[,1:2000]

table(Idents(normaldata))

tumordata<-tumordata[,1:2000]

table(Idents(tumordata))

###perform the cellchat analysis##

#devtools::install_github("sqjin/CellChat")

library(CellChat)

normal.input <- GetAssayData(normaldata, assay = "RNA", slot = "data") # normalized data matrix

labels <- factor(normaldata$Seurat_harmony,levels=levels(Idents(sce.mergeTEN)))

labels

meta <- data.frame(group = labels, row.names = rownames(normaldata@meta.data)) # create a dataframe of the cell labels

meta$group<-str_replace_all(meta$group,pattern = "/",replacement = "_")

meta$group <- factor(meta$group,levels=c("T cell","Epithelial","Smooth muscle cell","Fibroblasts","Endothelial cell","Macrophage","B cell","Lymphocyte","Plasma cell"))

cellchat_normal <- createCellChat(object = normal.input, meta = meta, group.by = "group")

saveRDS(cellchat_normal,file="./Cellchat/normalcellchat_1.rds")

tumor.input <- GetAssayData(tumordata, assay = "RNA", slot = "data") # normalized data matrix

labels <- factor(tumordata$Seurat_harmony,levels=levels(Idents(sce.mergeTEN)))

meta <- data.frame(group = labels, row.names = rownames(tumordata@meta.data)) # create a dataframe of the cell labels

meta$group<-str_replace_all(meta$group,pattern = "/",replacement = "_")

meta$group <- factor(meta$group,levels=c("T cell","Epithelial","Smooth muscle cell","Fibroblasts","Endothelial cell","Macrophage","B cell","Lymphocyte","Plasma cell"))

cellchat_tumor <- createCellChat(object = tumor.input, meta = meta, group.by = "group")

saveRDS(cellchat_tumor,file="./Cellchat/tumorcellchat_1.rds")

####prepare the deconvolution analysis gene signature####

rm(list=ls())

setwd("~/Documents_PC/scRNA-seq/Data")

normal_cellchat<-readRDS(file="./Cellchat/normalcellchat_1.rds")

tumor_cellchat<-readRDS(file="./Cellchat/normalcellchat_1.rds")

normal_cellchat@DB <- CellChatDB.human

gc()##释放内存

normal_cellchat <- subsetData(normal_cellchat) # subset the expression data of signaling genes for saving computation cost

#future::plan("multiprocess", workers = 2) # do parallel

#> Warning: [ONE-TIME WARNING] Forked processing ('multicore') is disabled

#> in future (>= 1.13.0) when running R from RStudio, because it is

#> considered unstable. Because of this, plan("multicore") will fall

#> back to plan("sequential"), and plan("multiprocess") will fall back to

#> plan("multisession") - not plan("multicore") as in the past. For more details,

#> how to control forked processing or not, and how to silence this warning in

#> future R sessions, see ?future::supportsMulticore

normal_cellchat <- identifyOverExpressedGenes(normal_cellchat)

normal_cellchat <- identifyOverExpressedInteractions(normal_cellchat)

normal_cellchat <- projectData(normal_cellchat, PPI.human)

normal_cellchat <- computeCommunProb(normal_cellchat, raw.use = TRUE)

# Filter out the cell-cell communication if there are only few number of cells in certain cell groups

normal_cellchat <- filterCommunication(normal_cellchat, min.cells = 1)

normal_cellchat <- computeCommunProbPathway(normal_cellchat)

normal_cellchat <- netAnalysis_computeCentrality(normal_cellchat, slot.name = "netP")

saveRDS(normal_cellchat,file="./Cellchat/normalcellchat_1.rds")

normal_cellchat<-readRDS("./Cellchat/normalcellchat_1.rds")

normal_cellchat <- aggregateNet(normal_cellchat)

groupSize <- as.numeric(table(normal_cellchat@idents))

groupSize

table(normal_cellchat@idents)

par(mfrow = c(1,2), xpd=TRUE)

netVisual_circle(normal_cellchat@net$count,arrow.size = 0.01, vertex.weight = groupSize, weight.scale = T, label.edge= F, title.name = "Number of interactions")

netVisual_circle(normal_cellchat@net$weight, arrow.size = 0.01,vertex.weight = groupSize, weight.scale = T, label.edge= F, title.name = "Interaction weights/strength")

mat <- normal_cellchat@net$count

#或者看weight

mat <- normal_cellchat@net$weight

dev.off()

####下面针对其中一个细胞亚型分析其对其他细胞的interaction的Ligand-receptor数量

mat2 <- matrix(0, nrow = nrow(mat), ncol = ncol(mat), dimnames = dimnames(mat))

mat2[1, ] <- mat[1, ]

netVisual_circle(mat2, vertex.weight = groupSize,arrow.size = 0.2, weight.scale = T, edge.weight.max = max(mat), title.name = rownames(mat)[1])

###查看具体的信号通路

df.net <- subsetCommunication(normal_cellchat)

#df.net_tumor <- subsetCommunication(tumor_cellchat)

levels(df.net$source)

pathways.show <- c("PDGF")

#table(df.net$pathway_name)

# Hierarchy plot

# Here we define `vertex.receive` so that the left portion of the hierarchy plot shows signaling to fibroblast and the right portion shows signaling to immune cells

#dev.new()

seq(1,4)

vertex.receiver = seq(1,4) # a numeric vector.

netVisual_aggregate(normal_cellchat, signaling = pathways.show, vertex.receiver = vertex.receiver,layout = "hierarchy")

netVisual_aggregate(normal_cellchat, signaling = pathways.show, vertex.receiver = vertex.receiver,layout = "chord")

#?netVisual_aggregate

#par(mfrow=c(1,1))

#netVisual_aggregate(normal_cellchat, signaling = pathways.show,vertex.receiver = vertex.receiver, layout = "circle")

netVisual_aggregate(normal_cellchat, signaling = pathways.show, layout = "circle")

netAnalysis_contribution(normal_cellchat, signaling = pathways.show)

####get specific cell

levels(normal_cellchat@idents)

netVisual_bubble(normal_cellchat, sources.use = c(1), targets.use = c(2:10), remove.isolate = FALSE)###epithelial

netVisual_bubble(normal_cellchat, sources.use = c(2), targets.use = c(8,9), remove.isolate = FALSE)##TNK

netVisual_bubble(normal_cellchat, sources.use = c(4), targets.use = c(1:3,5:9), remove.isolate = FALSE)##myeloid cell

#saveRDS(normal_cellchat,file="./Cellchat/normalcellchat_analysis.rds")

###We have run these in the workstation###

tumor_cellchat<-readRDS(file="./Cellchat/tumorcellchat_1.rds")

tumor_cellchat@DB <- CellChatDB.human

#gc()

tumor_cellchat <- subsetData(tumor_cellchat) # subset the expression data of signaling genes for saving computation cost

future::plan("multiprocess", workers = 4) # do parallel

tumor_cellchat <- identifyOverExpressedGenes(tumor_cellchat)

tumor_cellchat <- identifyOverExpressedInteractions(tumor_cellchat)

tumor_cellchat <- projectData(tumor_cellchat, PPI.human)

tumor_cellchat <- computeCommunProb(tumor_cellchat, raw.use = TRUE)

# Filter out the cell-cell communication if there are only few number of cells in certain cell groups

tumor_cellchat <- filterCommunication(tumor_cellchat, min.cells = 1)

tumor_cellchat <- computeCommunProbPathway(tumor_cellchat)

tumor_cellchat <- netAnalysis_computeCentrality(tumor_cellchat, slot.name = "netP")

saveRDS(tumor_cellchat,file="./Cellchat/tumorcellchat_1.rds")

tumor_cellchat<-readRDS("./Cellchat/tumorcellchat_1.rds")

tumor_cellchat <- aggregateNet(tumor_cellchat)

par(mfrow = c(2,2), xpd=TRUE)

groupSize <- as.numeric(table(tumor_cellchat@idents))

groupSize

table(tumor_cellchat@idents)

par(mfrow = c(1,2), xpd=TRUE)

netVisual_circle(tumor_cellchat@net$count,arrow.size = 0.01, vertex.weight = groupSize, weight.scale = T, label.edge= F, title.name = "Number of interactions")

netVisual_circle(tumor_cellchat@net$weight, arrow.size = 0.01,vertex.weight = groupSize, weight.scale = T, label.edge= F, title.name = "Interaction weights/strength")

mat <- tumor_cellchat@net$count

##mat <- tumor_cellchat@net$weight

#dev.off()

####下面针对其中一个细胞亚型分析其对其他细胞的interaction的Ligand-receptor数量

mat2 <- matrix(0, nrow = nrow(mat), ncol = ncol(mat), dimnames = dimnames(mat))

mat2[1, ] <- mat[1, ]

netVisual_circle(mat2, vertex.weight = groupSize,arrow.size = 0.2, weight.scale = T, edge.weight.max = max(mat), title.name = rownames(mat)[1])

###查看具体的信号通路

df.net <- subsetCommunication(tumor_cellchat)

#df.net_tumor <- subsetCommunication(tumor_cellchat)

levels(df.net$source)

pathways.show <- c("PDGF")

#table(df.net$pathway_name)

# Hierarchy plot

# Here we define `vertex.receive` so that the left portion of the hierarchy plot shows signaling to fibroblast and the right portion shows signaling to immune cells

#dev.new()

vertex.receiver = seq(1,4) # a numeric vector.

netVisual_aggregate(tumor_cellchat, signaling = pathways.show, vertex.receiver = vertex.receiver,layout = "hierarchy")

?netVisual_aggregate

par(mfrow=c(1,1))

netVisual_aggregate(tumor_cellchat, signaling = pathways.show, layout = "circle")

netAnalysis_contribution(tumor_cellchat, signaling = pathways.show)

####get specific cell

netVisual_bubble(normal_cellchat, sources.use = c(1), targets.use = c(2:10), remove.isolate = FALSE)###epithelial

netVisual_bubble(tumor_cellchat, sources.use = c(1), targets.use = c(2:10), remove.isolate = FALSE)###epithelial

netVisual_bubble(normal_cellchat, sources.use = c(2), targets.use = c(1,3:10), remove.isolate = FALSE)##TNK

netVisual_bubble(tumor_cellchat, sources.use = c(2), targets.use = c(1,3:10), remove.isolate = FALSE)##TNK

netVisual_bubble(normal_cellchat, sources.use = c(4), targets.use = c(1:3,5:9), remove.isolate = FALSE)##myeloid cell

netVisual_bubble(tumor_cellchat, sources.use = c(5), targets.use = c(1:4,6:10), remove.isolate = FALSE)##myeloid cell

#netAnalysis_signalingRole_network(normal_cellchat, signaling = c("PDGF"), width = 8, height = 2.5, font.size = 10)#,"VEGF"

# the slot 'netP' means the inferred intercellular communication network of signaling pathways

# Visualize the computed centrality scores using heatmap, allowing ready identification of major signaling roles of cell groups

netAnalysis_signalingRole_network(tumor_cellchat, signaling = c("PDGF"), width = 8, height = 2.5, font.size = 10)#,"VEGF"

netAnalysis_signalingRole_network(tumor_cellchat, signaling = c("IFN-II"), width = 8, height = 2.5, font.size = 10)

pdf(file="tumor1.pdf",width = 12, height = 10)

ht1 <- netAnalysis_signalingRole_heatmap(tumor_cellchat, pattern = "outgoing",height = 18)

ht2 <- netAnalysis_signalingRole_heatmap(tumor_cellchat, pattern = "incoming",height = 18)

ht1 + ht2

dev.off()

#normal_cellchat <- netAnalysis_computeCentrality(normal_cellchat, slot.name = "netP") # the slot 'netP' means the inferred intercellular communication network of signaling pathways

# Visualize the computed centrality scores using heatmap, allowing ready identification of major signaling roles of cell groups

netAnalysis_signalingRole_network(normal_cellchat, signaling = c("IGF"), width = 8, height = 2.5, font.size = 10)

netAnalysis_signalingRole_network(normal_cellchat, signaling = c("IFN-II"), width = 8, height = 2.5, font.size = 10)

pdf(file="normal1.pdf",width = 12, height = 10)

ht1 <- netAnalysis_signalingRole_heatmap(normal_cellchat, pattern = "outgoing",height = 18)

ht2 <- netAnalysis_signalingRole_heatmap(normal_cellchat, pattern = "incoming",height = 18)

ht1 + ht2

dev.off()

#saveRDS(normal_cellchat,file="./Cellchat/normalcellchat_analysis.rds")

#saveRDS(tumor_cellchat,file="./Cellchat/tumorcellchat_analysis.rds")

###we next compare the cell signaling pathways between the normal and tumor tissues##

data.dir <- './comparison'

dir.create(data.dir)

setwd(data.dir)

object.list <- list(normal = normal_cellchat, tumor = tumor_cellchat)

cellchat <- mergeCellChat(object.list, add.names = names(object.list))

#> Merge the following slots: 'data.signaling','net', 'netP','meta', 'idents', 'var.features' , 'DB', and 'LR'.

cellchat

gg1 <- compareInteractions(cellchat, show.legend = F, group = c(1,2))

gg2 <- compareInteractions(cellchat, show.legend = F, group = c(1,2), measure = "weight")

gg1 + gg2

par(mfrow = c(1,2), xpd=TRUE)

netVisual_diffInteraction(cellchat, weight.scale = T)###counts 差异

netVisual_diffInteraction(cellchat, weight.scale = T, measure = "weight")###weight的差异

gg1 <- netVisual_heatmap(cellchat)

#> Do heatmap based on a merged object

gg2 <- netVisual_heatmap(cellchat, measure = "weight")

#> Do heatmap based on a merged object

gg1 + gg2

gg1 <- rankNet(cellchat, mode = "comparison", stacked = T, do.stat = TRUE)

gg2 <- rankNet(cellchat, mode = "comparison", stacked = F, do.stat = TRUE)

gg1 + gg2

library(ComplexHeatmap)

#> Loading required package: grid

#> ========================================

#> ComplexHeatmap version 2.7.1.1010

#> Bioconductor page: http://bioconductor.org/packages/ComplexHeatmap/

#> Github page: https://github.com/jokergoo/ComplexHeatmap

#> Documentation: http://jokergoo.github.io/ComplexHeatmap-reference

#>

#> If you use it in published research, please cite:

#> Gu, Z. Complex heatmaps reveal patterns and correlations in multidimensional

#> genomic data. Bioinformatics 2016.

#>

#> This message can be suppressed by:

#> suppressPackageStartupMessages(library(ComplexHeatmap))

#> ========================================

i = 1

# combining all the identified signaling pathways from different datasets

pathway.union <- union(object.list[[i]]@netP$pathways, object.list[[i+1]]@netP$pathways)

ht1 = netAnalysis_signalingRole_heatmap(object.list[[i]], pattern = "outgoing", signaling = pathway.union, title = names(object.list)[i], width = 5, height = 6)

ht2 = netAnalysis_signalingRole_heatmap(object.list[[i+1]], pattern = "outgoing", signaling = pathway.union, title = names(object.list)[i+1], width = 5, height = 6)

draw(ht1 + ht2, ht_gap = unit(0.5, "cm"),height=unit(12, "cm"),width=unit(16, "cm"))

ht1 = netAnalysis_signalingRole_heatmap(object.list[[i]], pattern = "incoming", signaling = pathway.union, title = names(object.list)[i], width = 5, height = 6, color.heatmap = "GnBu")

ht2 = netAnalysis_signalingRole_heatmap(object.list[[i+1]], pattern = "incoming", signaling = pathway.union, title = names(object.list)[i+1], width = 5, height = 6, color.heatmap = "GnBu")

draw(ht1 + ht2, ht_gap = unit(0.5, "cm"),height=unit(12, "cm"),width=unit(16, "cm"))

ht1 = netAnalysis_signalingRole_heatmap(object.list[[i]], pattern = "all", signaling = pathway.union, title = names(object.list)[i], width = 5, height = 6, color.heatmap = "OrRd")

ht2 = netAnalysis_signalingRole_heatmap(object.list[[i+1]], pattern = "all", signaling = pathway.union, title = names(object.list)[i+1], width = 5, height = 6, color.heatmap = "OrRd")

draw(ht1 + ht2, ht_gap = unit(0.5, "cm"),height=unit(12, "cm"))

cellchat@idents

##Part III: Identify the upgulated and down-regulated signaling ligand-receptor pairs

netVisual_bubble(cellchat, sources.use = c(1), targets.use = c(4,7), comparison = c(1, 2), angle.x = 45)

netVisual_bubble(cellchat, sources.use = c(1), targets.use = c(2,3), comparison = c(1, 2), angle.x = 45)

###指定信号通路

netVisual_bubble(cellchat, sources.use = c(1), targets.use = c(4,7), signaling = c("MIF"), comparison = c(1, 2), angle.x = 90)

colnames(mat2)

##compare specific gene clusters### vlnplot

cellchat@meta$datasets = factor(cellchat@meta$datasets, levels = c("normal", "tumor")) # set factor level

plotGeneExpression(cellchat, signaling = "CXCL", split.by = "datasets", colors.ggplot = T)

plotGeneExpression(cellchat, signaling = "VEGF", split.by = "datasets", colors.ggplot = T)

plotGeneExpression(cellchat, signaling = "IGF", split.by = "datasets", colors.ggplot = T)

save.image(file="./Cell_chat.RData")
